# Informing Stewardship Measures in Canadian Food Animal Species through Integrated Reporting of Antimicrobial Use and Antimicrobial Resistance Surveillance Data—Part II, Application

**DOI:** 10.3390/pathogens10111491

**Published:** 2021-11-16

**Authors:** Agnes Agunos, Sheryl P. Gow, Anne E. Deckert, David F. Léger

**Affiliations:** 1 Center for Foodborne, Environmental and Zoonotic Infectious Diseases, Public Health Agency of Canada, Guelph, ON N1H 7M7, Canada; anne.deckert@phac-aspc.gc.ca (A.E.D.); david.leger@phac-aspc.gc.ca (D.F.L.); 2 Center for Foodborne, Environmental and Zoonotic Infectious Diseases, Public Health Agency of Canada, Saskatoon, SK S7N 5B4, Canada; sheryl.gow@phac-aspc.gc.ca

**Keywords:** integration, summarized reporting, antimicrobial use, antimicrobial resistance, stewardship, metric, indicator

## Abstract

Using the methodology developed for integrated analysis and reporting of antimicrobial use (AMU) and antimicrobial resistance (AMR) data, farm-level surveillance data were synthesized and integrated to assess trends and explore potential AMU and AMR associations. Data from broiler chicken flocks (n = 656), grower–finisher pig herds (n = 462) and turkey flocks (n = 339) surveyed by the Canadian Integrated Program for Antimicrobial Resistance Surveillance (CIPARS) at the farm-level (2015–2019) were used. The analyses showed a reduction in mean flock/herd level number of defined daily doses using Canadian standards (nDDDvetCA) adjusted for kg animal biomass that coincided with the decline in % resistance in the three species. This was noted in most AMU-AMR pairs studied except for ciprofloxacin resistant *Campylobacter* where resistance continued to be detected (moderate to high levels) despite limited fluoroquinolone use. Noteworthy was the significantly negative association between the nDDDvetCA/kg animal biomass and susceptible *Escherichia coli* (multispecies data), an early indication that AMU stewardship actions are having an impact. However, an increase in the reporting of diseases in recent years was observed. This study highlighted the value of collecting high-resolution AMU surveillance data with animal health context at the farm-level to understand AMR trends, enable data integration and measure the impact of AMU stewardship actions.

## 1. Introduction

Antimicrobials play an important role in the control of pathogens in food animal production; however, antimicrobial use (AMU) has contributed to the emergence of antimicrobial resistance (AMR) in zoonotic foodborne bacteria. In Canada, for example, the use of ceftiofur, a 3rd generation cephalosporin (3GC’s) for the prevention of neonatal infections in poultry has been associated with the emergence of ceftriaxone-resistant (CRO-R) *Salmonella* Heidelberg in people [1]. In recent years, numerous studies [2,3,4,5,6,7,8,9,10,11] have demonstrated linkages between AMU in food producing animals and AMR in select bacterial species. Linkages between AMU in livestock and AMR have also been reported in the European Union’s (EU) joint interagency antimicrobial consumption and resistance analysis (JIACRA) report [12]. These results emphasize that integration of AMU and AMR data from food producing animal species is essential towards understanding the larger ecology of AMR [13].

Surveillance of AMR and monitoring of AMU are components of the global call to contain AMR in the animal–environment–human interface [14,15,16]. Stewardship of AMU aligns with the global [14,15,16] and national [17] AMR strategies. As such, in 2019, enhanced veterinary oversight was implemented in Canada [18]. In parallel, the broiler chicken and turkey sectors also implemented AMU reduction strategies that aimed to progressively eliminate the use of certain classes of medically important antimicrobials [19,20]. In 2015, Step 1 eliminated the preventative use of category I antimicrobials such as 3GC’s and fluoroquinolones (FQ’s) [19,20]. Step 2, implemented in 2017, removed Category II antimicrobials (aminoglycosides [AMGL], macrolides [MACR], penicillins [PEN] and streptogramins [STRE]) for preventative use and step 3, the final step, focused on removal of preventative use of Category III antimicrobials, including bacitracins (BAC) and tetracyclines (TET) [19,20]. This approach to AMU stewardship aligns with the national [17] and global [14,15,16] recommendations. Information on AMU (e.g., trends over time, changes in total or class-specific quantity, profile of antimicrobial classes used, reasons for use) and AMR (e.g., trends over time, prevalence of multiclass resistance or susceptible isolates, resistance profiles), and how these surveillance data components relate to each other (i.e., AMU-AMR linkages) provides the “state of science” for AMU and AMR in animals and its potential implications in people, and thus is integral to antimicrobial stewardship and for measuring the impact of regulatory and voluntary changes in AMU.

Methodologies for development of AMU metrics (unit of measurement such as frequency or milligrams of antimicrobials) and indicators (metric in relation to a denominator) have substantially advanced in recent years as countries developed or strengthened their capacities for AMU monitoring [21,22] as part of an integrated AMU-AMR surveillance system [13] or as a requirement for regional and global data reporting [23,24]. The metric used is based on the study objectives whether it be monitoring of AMU over time, comparing AMU between species or geographical locations, benchmarking, or examining associations between AMU and AMR [21]. In Canada, AMU and AMR data have also been used to inform stakeholders on, for example, the impact of the first step of the poultry industry’s voluntary AMU reduction strategy was evaluated through the Canadian Integrated Program for Antimicrobial Resistance Surveillance (CIPARS) [19]. Additionally, CIPARS data have been used as a component of an AMR risk profiling exercise according to the Codex Alimentarius framework [25], an example of secondary uses of surveillance data. CIPARS monitors trends in AMU and AMR across the farm to fork continuum in select food animal species and in humans. CIPARS has a sentinel farm AMU and AMR surveillance component that involves the collection of AMU and AMR data from the same epidemiological unit. The CIPARS farm surveillance component provides AMU and AMR data to describe trends over time, differences between provinces/regions and variations between species. CIPARS previous work has highlighted the importance of using multiple AMU indicators for describing AMU exposure parameters [26,27], for understanding trends in both AMU and AMR in one CIPARS/FoodNet Canada Sentinel Site (descriptive only) [27], for characterizing flock distribution of AMU to identify high and low users of antimicrobials [28], and for using mg/kg animal biomass to study AMU and AMR associations [29]. To adapt to the rapidly evolving development of AMU metrics and indicators, CIPARS also explored how the current AMU indicators utilized in CIPARS reports relate to each other (i.e., weight based vs. dose-based) and how changes in denominator parameters affect levels of AMU (i.e., population correction unit based on animal population and average weight at treatment vs. kg animal biomass based on population and actual pre-slaughter live weights) [28]. These previous works addressed several AMU study objectives described in the literature. As well, the prior work informed refinements of surveillance methods and the development of an approach for within species and multispecies summarized analysis, data integration and reporting. In addition, as the animal sectors continue to implement voluntary and regulatory changes on AMU or modify their production practices, it is important to characterize the potential animal health implications of these changes. The purpose of this paper is to apply a proposed structured methodology for routine species-specific synthesis, integration and reporting of AMU and AMR, to utilize summarized AMU and AMR indicators for multispecies integration and for international comparisons, and to utilize the animal health component of the surveillance questionnaire for context to assess flock and herd health implications due to changes in AMU. This approach was built on our previous work [26,27,28,29,30] and is envisaged to be incorporated into CIPARS farm-level data analysis annually to track the progress and impact of AMU interventions (e.g., Steps 1 to 3 of the poultry AMU reduction strategies [19,20]) and stewardship actions [18]. As with other surveillance systems, this integrated AMU-AMR reporting approach could be progressively improved to adapt as integrated surveillance best practices evolve and to address stakeholder/data user feedback.

## 2. Results

During the study timeframe, the national sentinel farm sampling frame comprised of 696 broiler chicken flocks (Mean 139 flocks/year), 462 GF pig herds (mean 92 herds/year) and 339 turkey flocks (mean 85 flocks/year). On average, there were 17 poultry and 20 swine sentinel veterinary practices that participated in the program. The following key findings were synthesized using a structured approach for AMU-AMR species level and multispecies integration. For the first time, the exploratory AMU indicator (nDDDvetCA/kg animal biomass) (described in reference [28]) for total and class-specific AMU and the corresponding “composite” AMR outcomes (susceptible and multiclass resistance and homologous resistances) were utilized. Animal health data (syndromic and preventive health) were used as contextual information to understand the potential implications of the changes in AMU trends.

### 2.1. AMU-AMR Integration, Species Level

#### 2.1.1. Broiler Chickens

##### AMU and AMR Temporal Trends

**Figure 1:**
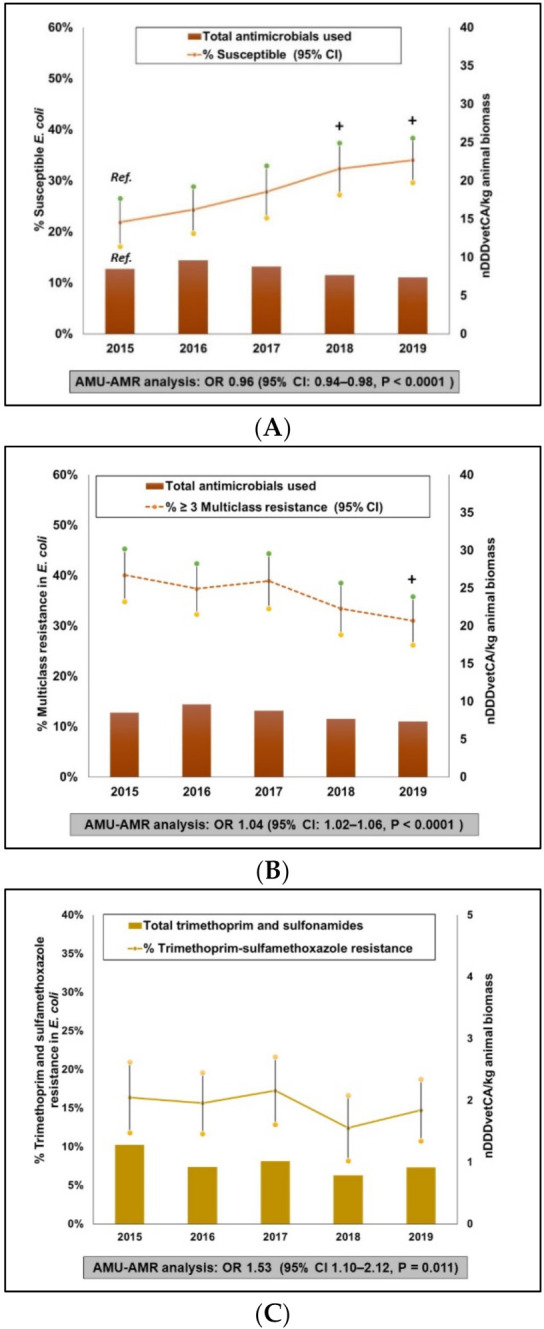

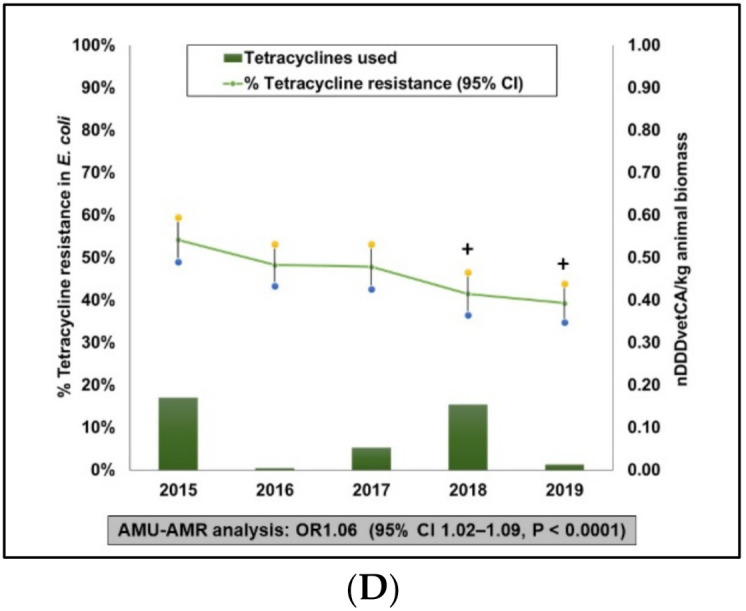
Integration of antimicrobial use and antimicrobial resistance in broiler chickens, 2015 to 2019. Total antimicrobials used—Susceptible (i.e., isolates that exhibited reduced or intermediate susceptibility to all antimicrobials tested) (**A**). Total antimicrobials used—multiclass resistance (i.e., isolates that exhibited resistance to antimicrobials from at least three different antimicrobial classes) (**B**). Trimethoprim and sulfonamides used—Trimethoprim and sulfamethoxazole resistance (**C**). Tetracycline used—Tetracycline resistance (**D**). + Significant (*p* ≤ 0.05) difference in AMR compared to the referent year (2015, labelled as Ref in (**A**)). OR–Odds Ratio, 95%CI–95% confidence intervals. Data points for AMU are flock-level mean nDDDvetCA/kg animal biomass and for AMR, these are annual mean % resistance with 95% confidence intervals and adjusted for clustering at the flock-level to account for multiple samples per flock. AMU-AMR associations were evaluated using mixed effects logistic regression models.

AMU and AMR data are shown in Figure 1. The annual flock level mean nDDDvetCA/kg animal biomass and mean resistance or susceptible (95% CI) adjusted for clustering at the flock-level are presented. A non-significant downward trend in flock-level total nDDDvetCA/kg animal biomass was observed. A 14% decrease in nDDDvetCA/kg animal biomass was detected between 2015 and 2019. During the same timeframe, susceptible *E. coli* significantly increased by 12% (*p* < 0.0001). Parallel to this finding was the significant decrease in multiclass resistant *E. coli* by 9% (*p* = 0.01). The 10 most frequently occurring antimicrobial class patterns are summarized in Supplementary Materials S1, Table S1. For the homologous AMR indicators, low-level CRO-R continued to be detected, however it significantly decreased between 2015 and 2019 by 5% (*p* = 0.03). During the last 2 years of surveillance, there has been no reported use of 3GCs. The use of AMGL and aminocyclitol (AMCL) peaked in 2018, however in 2019, the use decreased back to levels similar to 2015 in 2019. Concurrently, gentamicin resistance (GEN-R) has remained at a moderate but relatively stable prevalence between 2015 and 2019 (19% to 21%). Trimethoprim and sulfamethoxazole resistance (SXT-R) fluctuated during the study timeframe with a slight non-significant increase of 3% between 2018 and 2019. In parallel, a nonsignificant decrease in trimethoprim and sulfonamide (TMPS) use by 29% (1.3 to 0.9 nDDDvetCA/kg animal biomass) between 2015 and 2019 was noted. Tetracycline resistance (TET-R) prevalence significantly decreased by 15% (*p* < 0.0001) between 2015 and 2019 while TET use fluctuated but no significant temporal changes were detected.

Ciprofloxacin resistant *Campylobacter* fluctuated during the study timeframe. A significant increase of 9% (*p* = 0.05) was noted between 2018 and 2019, but corresponding FQ use was rarely reported (0.01 nDDDvetCA/kg animal biomass), with only one flock in 2018 having reported any FQ use. Azithromycin resistant (AZM-R) *Campylobacter* significantly decreased by 17% (*p* = 0.002) between 2015 and 2019. The decrease in AZM-R corresponded with a statistically significant decrease in macrolide (MACR) use during the first 3 years of the study from 0.11 to 0.02 nDDDvetCA MACR/kg animal biomass, but MACR use reverted to 2015 level (0.11) in 2018, and then dropped to zero use in 2019. An alternate AMU predictor for AZM-R was MLSB (the combined MACR,-lincosamides [LINC] and STRE B; virginiamycin product available in Canada contains both STRE A and B). For this combined class, the nDDDvetCA increased in 2016 and 2017 (>2 nDDDvetCA/kg animal biomass) compared to 2015 (1.24 nDDDvetCA/kg animal biomass). However, the levels dropped significantly (*p* < 0.0001) from 1.24 in 2015 to 0.24 nDDDvetCA MLSB/kg animal biomass in 2019.

##### AMU-AMR Association

Combinations of five AMR outcomes in *E. coli* and the corresponding AMU (total or class-specific) were investigated. Mixed effects logistic regression analysis adjusted for year showed a statistically significant negative association between total nDDDvetCA/kg animal biomass and susceptible *E. coli* (OR 0.96, 95% CI 0.94–0.98, *p* < 0.0001). Logistic regression plots (not shown) indicated that the predicted probability of detecting susceptible isolates was lower in isolates from flocks that were exposed to higher quantities of antimicrobials using nDDDvetCA/kg animal biomass as a measurement. In parallel, a significant positive association was observed between total nDDDvetCA/kg animal biomass and multiclass resistance (OR 1.04, 95% CI 1.02–1.06, *p* < 0.0001). Other significant positive associations were noted for the following pairs: SXT-R and TMPS AMU (1.53, 95%CI 1.10–2.12, *p* = 0.01), and TET-R and TET AMU (OR = 1.06, 95% CI 1.02–1.09, *p* = 0.001). No statistically significant association between GEN-R and AMIN/AMCL AMU was detected (not shown). Two AMR outcomes in Campylobacter and the corresponding composite and class-specific AMU were investigated. The association for the AZM-R and MACR use pair as well, AZM-R and MLSB yielded nonsignificant associations. The association between CIP-R and FQ AMU was not determined because there was only 1 report of FQ use during the study timeframe.

##### AMU-Flock Health

During the study timeframe, mortality (Supplementary Materials S2) remained stable over time (mean 3.92%, range 0.43–23.83%). The highest reported mortality was experienced by one flock in 2015 and it was noted that this mortality was brooding temperature related. 

**Figure 2:**
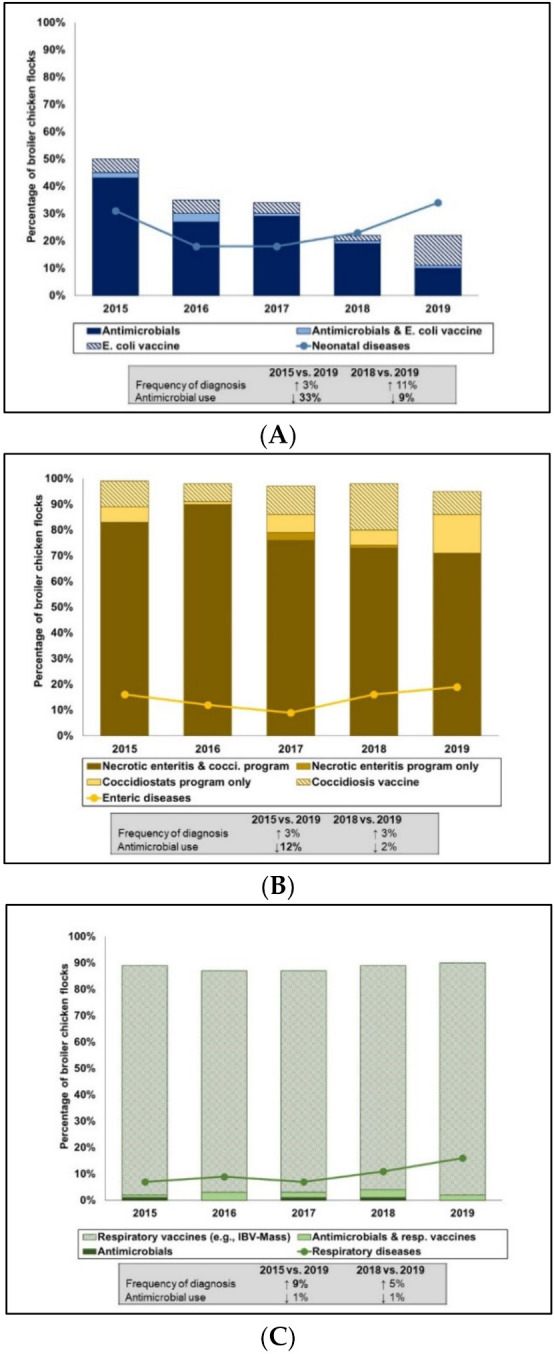
Integration of disease syndromes reported and relevant antimicrobial use using count-based indicator in broiler chickens, 2015 to 2019. Neonatal diseases comprised of yolk sac infection and early septicemia (**A**). Enteric diseases comprised of necrotic enteritis, coccidiosis and nonspecific enteric diseases (**B**). Respiratory Diseases comprised of airsacculitis and manifestations/gross lesions of respiratory infections (**C**). Respiratory vaccines in C were largely Massachusetts type of Infectious Bronchitis Virus vaccine. Values in bold fonts within the text boxes indicates significant change (*p* ≤ 0.05) and the arrows signify the direction of the shift.

Integrated AMU–animal health data synthesized from the AMU and animal health sections of the questionnaires (e.g., disease syndromes or etiologic agents where responses were positive or likely positive, vaccination programs specific to the disease, and other preventive health programs) are shown in Figure 2A–C. Notable shifts in AMU and diseases diagnosed during the study timeframe and changes between the last 2 years of surveillance are demonstrated in the figures. Neonatal diseases such as yolksacculitis/omphalitis with septicemic sequalae, usually associated with avian pathogenic *E. coli* (APEC) significantly dropped from 30% in 2015 to 18% in 2016 (*p* = 0.03). Another significant drop of 18% between 2015 and 2017 (*p* = 0.02) was also detected. There was an increase in neonatal disease from 23% to 34% between 2018 and 2019, respectively, however, the increases were not statistically significant compared to 2015. This trend coincided with a statistically significant (*p* < 0.0001) drop in reported AMU at the hatchery and early brooding stages (first 2 weeks) from 43% in 2015 to 5% in 2019. A small proportion of flocks used both *E. coli* vaccines and antimicrobials to prevent or control *E. coli* associated infections. Though not statistically significant, vaccination against *E. coli* trended upwards from 5% (2015) to 11% (2019) during the study timeframe. The commercially available *E. coli* vaccine is an O78 strain that has a claim for prevention of *E. coli* associated infections.

The percentage of flocks with reported diagnosis of enteric diseases (i.e., necrotic enteritis, coccidiosis and nonspecific enteritis) fluctuated over time. There was an increase from 9% in 2017 to 16% in 2018 and then to 19% in 2019 (Figure 2B), however these increases were not statistically significant compared to 2015. The most common intervention against enteric diseases was the implementation of both a coccidiosis (cocci) and a necrotic enteritis (NE) preventive program. This practice significantly (*p* = 0.04) decreased by 12% between 2015 and 2019. This reduction coincided with a significant 9% (*p* = 0.07) increase between 2015 and 2019 in the number of flocks on coccidiostat programs alone (i.e., no antimicrobials used for NE prevention, such as the flocks marketed as “Raised Without Antibiotics” [RWA]). Flocks that used coccidiosis vaccine alone (no AMU) fluctuated over time and reached a peak of 18% in 2018. However, this dropped by 9% the following year. None of these changes were statistically significant. Detailed control programs for the prevention and control of coccidiosis including the use of coccidiosis vaccines and the use of coccidiostats either continuous (also known as a straight program, using one coccidiostat throughout the growing period), shuttle (also known as a dual program, using two or more coccidiostats during the growing period) or as a bioshuttle program (use of coccidiosis vaccine and coccidiostats) can be found in Figure S1. Necrotic enteritis programs that entailed the use of 1, 2 or ≥3 antimicrobials during the growing period are summarized in Figure S2.

Respiratory diseases (i.e., classified as confirmed or probably positive for airsacculitis in the questionnaire) significantly increased by 9% (*p* = 0.021) between 2015 and 2019. AMU for managing respiratory diseases was relatively uncommon (1–3%). Respiratory vaccines against viral agents such as field challenge of Infectious Bronchitis Virus that could complicate bacterial infections (i.e., largely APEC) appeared to be a consistent preventive health approach with up to 88% of flocks vaccinated at least once (i.e., at the hatchery level, on farm or both).

Miscellaneous bacterial diseases (data not shown) comprised of mid to late-stage septicemia (colibacillosis sequelae), staphylococcal tenosynovitis/osteomyelitis, vertebral canal osteomyelitis caused by *Enterococcus cecorum* and salmonellosis were reportedly experienced by an average of 9% of the flocks each year. There were no remarkable temporal trends in AMU for treating these diseases.

##### Overall AMU, AMR and Flock Health Situation in Broiler Chickens

Various information collected from the farm questionnaire and AMR data enabled assessment of the current status of AMU and AMR in broiler chickens. AMU trended downwards and although the decrease was not significant, this trend corresponded with a significant increase in susceptible *E. coli* and decreased multiclass resistant *E. coli*. These results are an early indication that the composition of the bacterial population (multiclass resistant towards more susceptible population) in broiler chickens has shifted during the study timeframe. Low-level CRO-R *E. coli* continued to be detected, and there was a significant increase in CIP-R *Campylobacter*, although corresponding AMU (3GCs, FQs) was limited. In the face of reduced AMU in the broiler chicken sector, the diagnosis of common diseases of broilers has increased. Over the study period there were other preventive health approaches implemented, for example, replacement of hatchery level and early brooding AMU with vaccination against *E. coli*. Additionally, there was a shift from NE and cocci programs based on antibiotics (including BAC, STRE, orthosomycins) plus coccidiostats (e.g., ionophores such as narasin and monensin, chemical or synthetic coccidiostats such as amprolium, nicarbazine, robenidine) to the sole use of chemical or synthetic coccidiostats (i.e., allowed in RWA production system) as more flocks transitioned to RWA production or reduced AMU production programs over time.

#### 2.1.2. GF Pigs

##### AMU and AMR Temporal Trends

**Figure 3:**
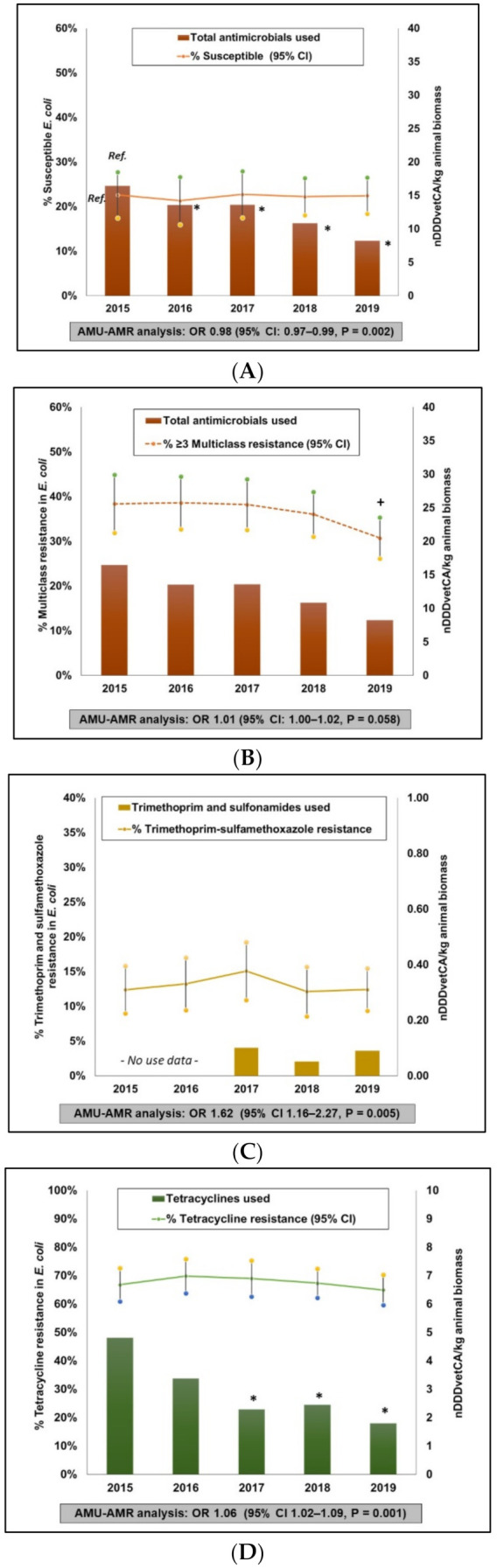

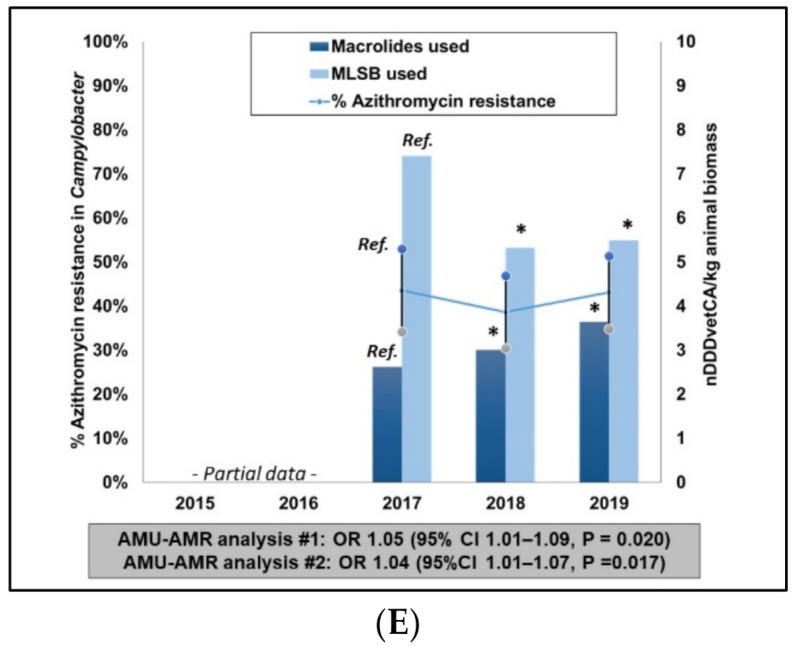
Integration of antimicrobial use and antimicrobial resistance in grower-finisher swine herds, 2015 to 2019. Total antimicrobials used—Susceptible (i.e., isolates that exhibited reduced or intermediate susceptibility to all antimicrobials tested) (**A**). Total antimicrobials used—Multiclass resistance (i.e., isolates that exhibited resistance to antimicrobials from at least three different antimicrobial classes) (**B**). Trimethoprim and sulfonamides used—Trimethoprim and sulfamethoxazole resistance (**C**). Tetracycline used—Tetracycline resistance (**D**). Analysis #1: Macrolides used—Azithromycin resistance and Analysis #2: Macrolides, lincosamides and streptogramin B use—Azithromycin resistance (**E**). * Significant (*p* ≤ 0.05) difference in AMU compared to the referent year (2015, labelled as Ref. in (**A**)). + Significant (*p* ≤ 0.05) difference in AMR compared to the referent year (2015). OR—Odds Ratio, 95%CI—95% confidence intervals. Data points for AMU are herd-level mean nDDDvetCA/kg animal biomass and for AMR, these are annual mean resistance percentages with 95% confidence intervals adjusted for clustering at the herd-level to account for multiple samples per herd. AMU-AMR associations were evaluated using mixed effects logistic regression models.

Combined AMU-AMR data are shown in Figure 3. As with broiler chickens, AMU values are the annual herd-level mean nDDDvetCA/kg animal biomass and resistance percentages (95% CI) that were adjusted for clustering at the herd level. The total nDDDvetCA/kg animal biomass decreased over time, with a significant (*p* = 0.008) drop of 50% between 2015 and 2019. While trends in susceptible *E. coli* remained stable over time (22 to 23%), multiclass resistant *E. coli* decreased significantly (*p* = 0.04) by 8% in 2019 compared to 2015. Antimicrobial class resistance profiles are summarized in Table S2. As for the homologous AMR outcomes, low prevalence of CRO-R (0 to 2%) and GEN-R (1 to 2%) were detected and the corresponding use of 3GC’s (2017–2019) and AMGL (2015), respectively, were relatively low. Trimethoprim and sulfamethoxazole resistance remained stable; ranging from 12% to 15% during the study timeframe, with no significant temporal changes. Corresponding TMPS use was reported between 2017 and 2019 and similar to the SXT-R, no significant temporal changes were detected. It is important to note that quantitative data on antimicrobials administered via injection and drinking water in GF pigs were available only after 2017. Tetracycline resistance reached a peak of 70% in 2016, however since then, the percentage of TET-R has trended down to 65%, though this decrease was not significant. This trend paralleled the trend in TET use, where a significant (*p* = 0.006) drop by 62% between 2015 and 2019 was observed. 

AMR testing for *Campylobacter* started in 2017 in GF pigs. A non-significant increase in CIP-R *Campylobacter* from 8% in 2017 to 12% in 2019 was noted. Fluoroquinolones were reportedly only used in 2017 (<0.1 nDDDvetCA/kg animal biomass). A high (40% to 44%) relatively stable prevalence of AZM-R *Campylobacter* were detected along with a corresponding stable use of MACR (3 to 4.2 nDDDvetCA/kg animal biomass) during the study timeframe.

##### AMU-AMR Association

Similar to broiler chickens, mixed effects logistic regression analysis showed statistically significant negative associations between total nDDDvetCA/kg animal biomass and susceptible *E. coli* (OR 0.98, 95% CI, 0.96–0.98, *p* = 0.002). Though total nDDDvetCA/kg animal biomass and multiclass resistant *E. coli* yielded an OR of 1.11 (95% CI, 1.04–1.19), the association was not significant. The association between GEN-R and AMGL use and CRO-R and 3GC use were not determined since relatively few herds were reporting use of AMGL (1 year) and 3GCs (3 years but at very low levels) with relatively very low resistance prevalence. As for SXT-R and TMPS use, in years where data from feed, water and injection were available (2017 to 2019), a significant association was observed (OR 1.62, 95% CI, 1.16–2.27, *p* = 0.004) between AMU and AMR. Tetracycline resistance and TET use, similarly, yielded a statistically significant association (OR 1.06, 95% CI, 1.02–1.09, *p* = 0.001) between AMU and AMR.

The association between CIP-R *Campylobacter* and FQ use was not determined due to low prevalence of AMR and AMU for this pair of interest. As for AZM-R and MACR use in years with complete (feed, water and injection) AMU data (2017 to 2019), a significant association was observed (OR 1.05, 95%CI 1.01–1.09, *p* = 0.02) between AMU and AMR. The association between AZM-R and the alternate predictor variable, MLSB, yielded similar findings (OR 1.04, 95% CI, 0.01–1.07, *p* = 0.02) between AMU and AMR.

##### AMU-Herd Health

**Figure 4:**
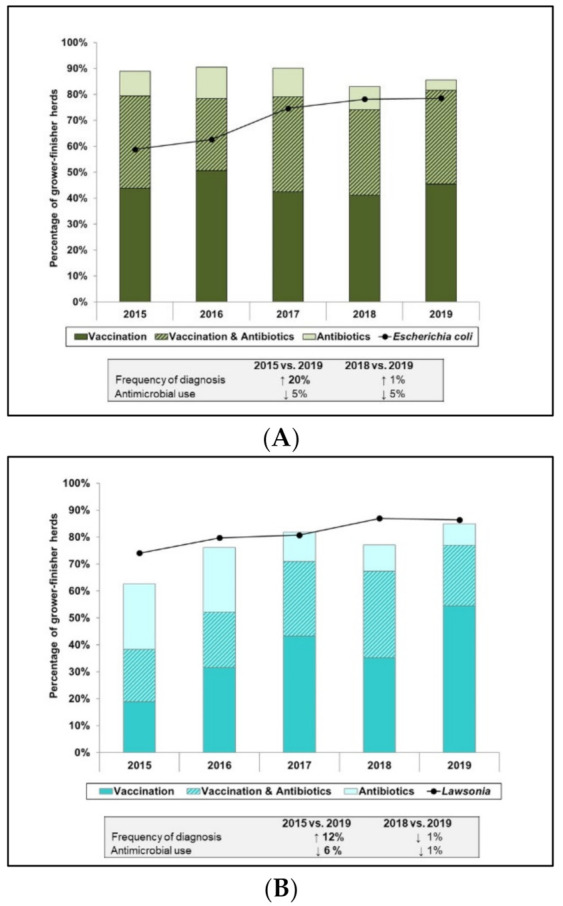

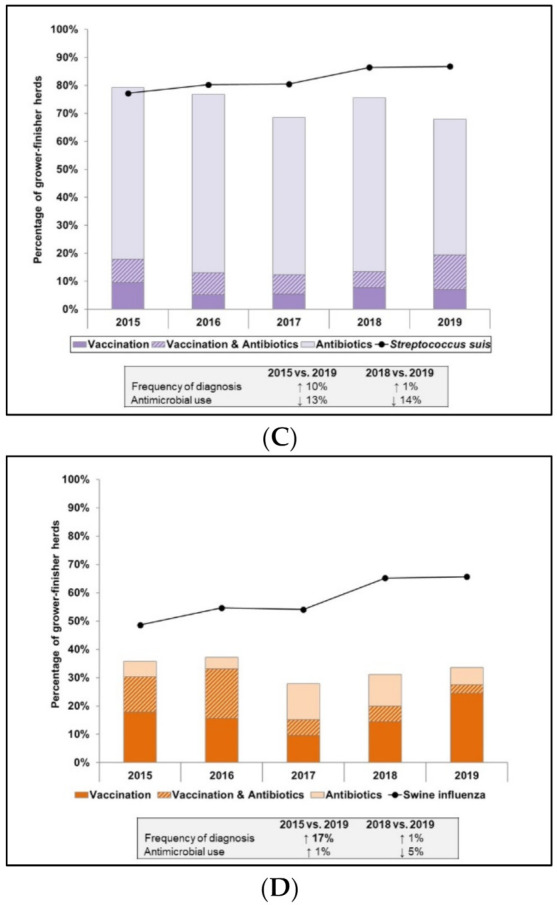
Integration of disease syndromes reported and relevant antimicrobial use using count-based indicator in grower finisher pigs, 2015 to 2019. *Escherichia coli* (**A**). *Lawsonia* spp. (**B**). *Streptococcus suis* (**C**). Swine Influenza Virus/Secondary bacterial infection (**D**). Antimicrobial use in D was intended for secondary bacterial diseases associated with Swine Influenza Virus infections/field challenge. Values in bold fonts within the text boxes indicates significant change (*p* ≤ 0.05) and the arrows signify the direction of the shift.

Mean GF herd mortality during the study timeframe was 2.35% (range: <1 to 11%) (Supplementary Materials S2) and there were no significant temporal changes noted. As with the broiler chicken section, integrated AMU–animal health data synthesized from the AMU and animal health sections of the questionnaire were descriptively assessed. Select disease etiologic agents in GF pigs and relevant herd health variables are shown in Figure 4. 

The reporting of *E. coli* significantly increased from 59% in 2015 to 79% in 2019 (*p* = 0.007). The use of vaccines and antimicrobials however, was relatively stable over the same period with between 4 and 12% of herds using antimicrobials, between 41 and 51% of herds using vaccines and between 28 and 37% of herds using both antimicrobials and vaccines to treat or control this disease. Efficacy of both autogenous and commercial vaccines is dependent on the specific strains of *E. coli* that are present on the farm, since these can vary.

There was a significant increase in reporting of *Lawsonia* from 74% in 2015 to 86% in 2019 (*p* < 0.05). There was a corresponding decrease in the use of antimicrobials to treat or control this disease from 24 to 8% over the same time period (*p* = 0.004). In addition, the number of GF herds that reportedly used vaccines for *Lawsonia* significantly increased from 19% in 2015 (*p* < 0.0010) and 35% in 2018 (*p* = 0.008) to 55% in 2019. The number of herds using both vaccines and antimicrobials to treat or control *Lawsonia* did not significantly differ and ranged from 19% in 2015 to 32% in 2018 and 22% in 2019.

*Streptococcus suis* was very common on GF herds with 77% and 87% of herds that reportedly diagnosed with this disease in 2015 and 2019, respectively. The percentage of herds with reported AMU to treat or control *Streptococcus suis* decreased from 61% in 2015 and 62% in 2018 to 48% in 2019, although these changes were not significant. The percentage of herds using vaccines or both antimicrobials and vaccines for *Streptococcus suis* was relatively stable over this time period and ranged from 5% to 10% and 6% to 12%, respectively. The lack of an available, efficacious commercial vaccine for *Streptococcus suis* and the ubiquitous nature of this organism on pig farms, limits the potential for AMU reduction.

The percentage of GF herds with reported Swine Influenza significantly increased from 49% in 2015 to 66% in 2019 (*p* = 0.26). Over the same time period, the percentage of herds that reportedly used vaccines to control Swine Influenza decreased from 18% in 2015 to 10% in 2017 but subsequently increased to 24% in 2019, although these changes were not significant. There were no significant differences in antibiotic use for secondary bacterial infections as sequelae to Swine Influenza Virus infections, which ranged from 5% of herds in 2015 to 13% in 2017 and 6% in 2019. However, there was a significant decrease in herds using both antimicrobials and vaccines (13% in 2015 to 3% in 2019, *p* = 0.039) for this disease, and therefore overall use trended down. As in other species, the wide range of Swine Influenza Virus strains and the lack of cross-protection can limit the effectiveness of Swine Influenza vaccines, however current initiatives to develop regional autogenous Swine Influenza vaccines appear promising.

Other common diseases/etiologic agent reported on GF pig farms included erysipelas, *Haemophilus parasuis, Mycoplasma* spp., and Porcine Circovirus-Associated Disease (PCVAD). The use of either antimicrobials or vaccines for these diseases was stable between 2015 and 2019 (data not shown). The use of both antimicrobials and vaccines to control these diseases on the same farm was not uncommon, but a general non-significant downward trend was observed from 2015 to 2019. In 2019, the percentage of herds using this practice varied depending on the disease syndrome/etiologic agent: erysipelas (28%), *Mycoplasma* spp. (26%), *Heamophilus parasuis* (11%), and PCVAD (4%).

##### Overall AMU, AMR and Herd Health Situation in GF Pigs

Similar to chickens, data collected through the questionnaires and AMR data from collected fecal samples enabled the assessment of the current AMU-AMR status in GF pigs. Susceptible *E. coli* was relatively stable over the study period but a decreasing trend in multiclass resistance was noted which mirrored the declining trend in total AMU. Noteworthy was the increasing trend in MACR use (and MLSB) that corresponded with relatively high AZM-R levels. Antimicrobials and vaccines were commonly used to manage the economically important diseases (largely systemic in nature) but the limited cross-protection of available vaccines against the field strains (e.g., *Streptococcus* spp., Swine Influenza Virus) has been a challenge for AMU reduction.

#### 2.1.3. Turkeys

##### AMU and AMR Temporal Trends

**Figure 5:**
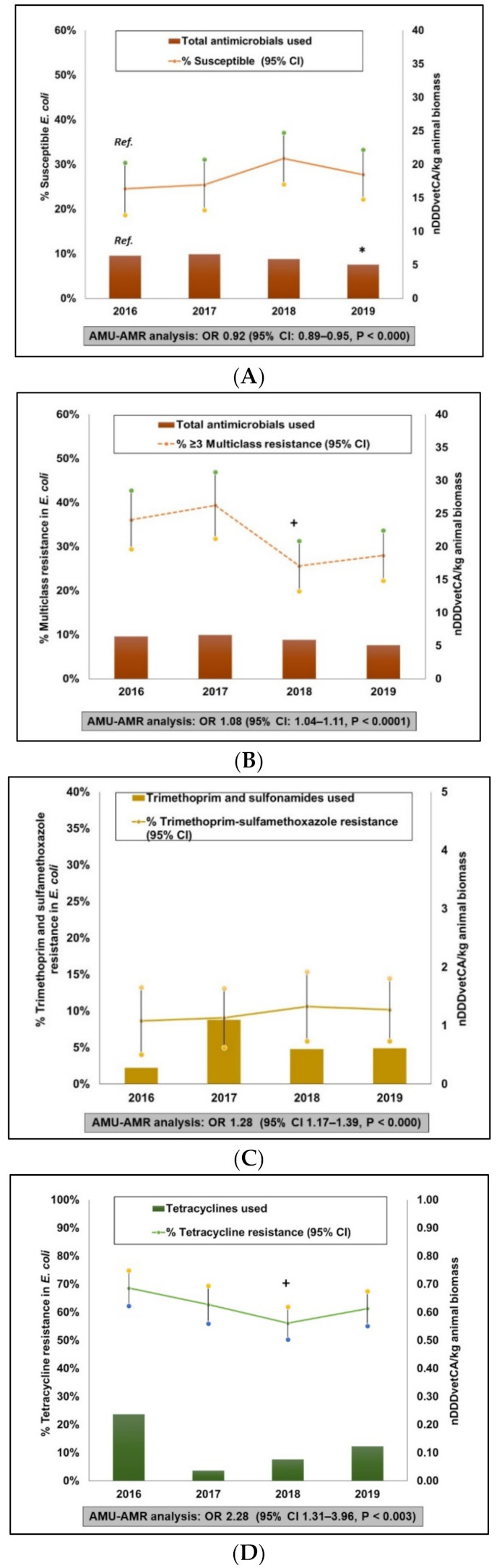
Integration of antimicrobial use and antimicrobial resistance in turkeys, 2016 to 2019. Total antimicrobials used—Susceptible (i.e., isolates that exhibited reduced or intermediate susceptibility to all antimicrobials tested) (**A**). Total antimicrobials used—multiclass resistance (i.e., isolates that exhibited resistance to antimicrobials from at least three different antimicrobial classes) (**B**). Trimethoprim and sulfonamides used—Trimethoprim-sulfamethoxazole resistance (**C**). Tetracycline used—Tetracycline resistance (**D**).* Significant (*p* ≤ 0.05) difference in AMU compared to the referent year (2016, labelled as Ref. in (**A**)). + Significant (*p* ≤ 0.05) difference in AMR compared to the referent year (2016). OR—Odds Ratio, 95%CI—95% confidence intervals. Data points for AMU are flock-level mean nDDDvetCA/kg animal biomass and for AMR, these are annual mean resistance percentages with 95% confidence intervals adjusted for clustering at the flock-level to account for multiple samples per flock. AMU-AMR associations were evaluated using mixed effects logistic regression models.

Combined AMU-AMR data are shown in Figure 5. Though not significant, a downward trend in total nDDDvetCA/kg animal biomass was observed. Overall, a 21% decrease was detected between 2016 and 2019. There were no significant temporal changes in susceptible *E. coli* but multiclass resistant *E. coli* significantly (*p* = 0.02) dropped by 10% between 2016 and 2018, however this resistance increased by 2% in 2019. Antimicrobial class resistance profiles are summarized in Table S3. Low prevalence of CRO-R (1% to 2%) continued to be detected though there was no reported use of 3GCs during the study timeframe. Aminoglycosides use fluctuated over time and in 2019, it reverted to near the 2015 level (0.016 nDDDvetCA/kg animal biomass). In parallel, GEN-R trended downward and significantly decreased in the last 2 years of the study timeframe, by 7% between 2016 and 2018 (*p* = 0.042) and by 11% between 2016 and 2019 (*p* = 0.002). Trimethoprim and sulfonamides use fluctuated over time, but no substantial changes were observed during the study timeframe while SXT-R remained relatively stable between 9% and 11%. During the study timeframe, a nonsignificant change in TET use was observed (2016 and 2019), however TET use fluctuated over time while TET-R significantly (*p* = 0.005) decreased by 12% between 2015 and 2018, but subsequently increased marginally by 5% the following year (2019).

Ciprofloxacin resistant *Campylobacter* remained at a moderately high prevalence (>20%) with a 13% increase in resistance between 2016 and 2019. Despite rising CIP-R in *Campylobacter* there was only one flock with reported FQ use (2018) during the study timeframe. Initially low prevalence of AZM-R *Campylobacter* was detected (1% in 2016) but AZM-R significantly (*p* = 0.01) increased by 4% the following year. Subsequently AZM-R dropped by 7% in 2018 and continued to drop by another 3% in 2019. Macrolide use was reported during the first 2 years of the study timeframe, with a cumulative total of 0.11 nDDDvetCA/kg animal biomass; there was no MACR use reported in 2018 and 2019. The alternate AMU variable, MLSB significantly (*p* < 0.0001) dropped from 3 nDDDvetCA/kg animal biomass to 0.20 nDDDvetCA/kg animal biomass in 2019.

##### AMU-AMR Association

Similar to broiler chickens and GF pigs, mixed effects logistic regression analysis showed statistically significant associations (*p* ≤ 0.05) between total nDDDvetCA/kg animal biomass and susceptible *E. coli* (OR 0.92, 95% CI, 0.89–0.95, *p* < 0.0001). Complementary to this finding, there was a significant association between total nDDDvetCA/kg animal biomass and multiclass resistant *E. coli* (OR 1.08, 95% CI 1.04–1.11, *p* < 0.0001). Other significant associations detected were between SXT-R and TMPS use (OR 1.28, 95%CI 1.17–1.39, *p* < 0.0001) and between TET-R and TET use (or 2.28, 95%CI 1.31–3.96, *p* = 0.003). Associations between CIP-R and fluoroquinolones use and between AZM-R and macrolides use (and MLSB) were not determined. 

##### AMU-Flock Health

**Figure 6:**
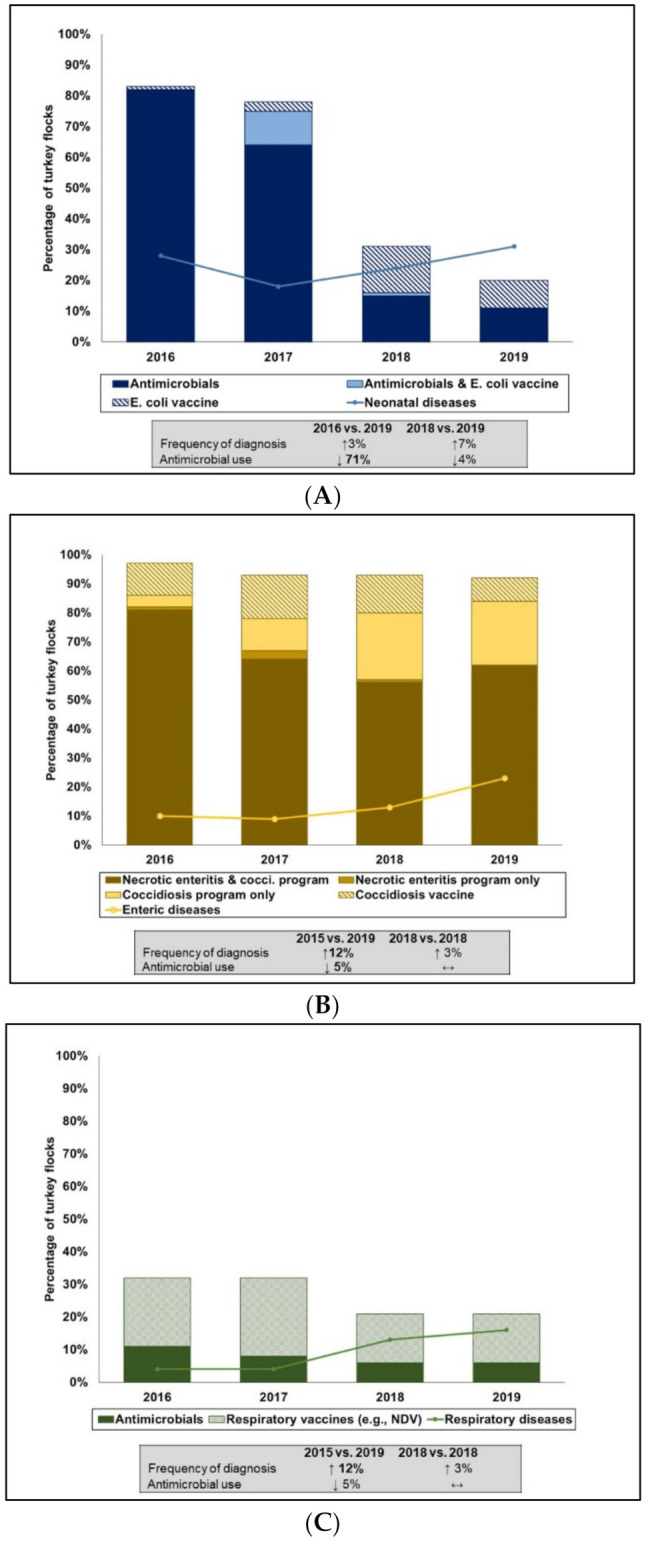
Integration of disease syndromes reported and relevant antimicrobial use using count-based indicator in turkeys, 2016 to 2019. Neonatal diseases comprised of yolk sac infection and early septicemia (**A**). Enteric diseases comprised of necrotic enteritis, coccidiosis and nonspecific enteric diseases (**B**). Respiratory Diseases comprised of airsacculitis and manifestations/gross lesions of respiratory infections (**C**). Respiratory vaccines in C were Newcastle Disease Virus administered one to three times during the growing period. Values in bold fonts within the text boxes indicates significant change (*p* ≤ 0.05) and the arrows signify the direction of the shift.

During the study timeframe, mortality (Supplementary Materials S2) remained stable over time (mean: 6.32%, 95%CI: 0.53–32.72%). The percentage of flocks reportedly diagnosed with disease syndromes, AMU intended for that clinical condition, and relevant animal health programs were combined in Figure 6. As shown in Figure 6A, neonatal diseases dropped from 28% to 18% between 2016 and 2017 but increased to 24% in 2018 and 31% in 2019; these changes were non-significant. As for the AMU, there was a significant (*p* < 0.0001) drop in reported AMU at the hatchery and early brooding stages (first 2 weeks) from 82% (2016) to 11% (2019). Flocks that used an *E. coli* vaccine and antimicrobials to manage APEC-associated infections peaked at 11% in 2017 but dropped to 1% in 2018. Vaccination against *E. coli* significantly (*p* = 0.02) increased from 1% in 2016 to 15% in 2018.

The percentage of flocks reportedly experienced enteric diseases gradually increased from 0% to 13% between 2016 and 2018, then increased significantly (*p* = 0.03) to 23% in 2019 compared to 2016 levels (Figure 6B). Similar to broiler chickens, flocks that used NE-cocci preventive programs were the most common intervention against enteric diseases overall, but flocks using both programs significantly (*p* = 0.015) dropped from 81% in 2016 to 62% in 2019. This coincided with a significant (*p* = 0.003) increase in the number of flocks on a coccidiostats program alone from 4% in 2016 to 22% in 2019. Flocks that used coccidiosis vaccine alone fluctuated over time, with a peak of 15% in 2017 and a drop to 8% in 2019. Changes detected were non-significant. More detailed control programs for NE and cocci are found in Figure S3A,B.

Respiratory diseases significantly (*p* = 0.024) increased from 4% in 2016 to 16% in 2018 but turkey flocks with reported AMU for this disease decreased (non-significant) from 11% to 6% during the study timeframe. The use of respiratory vaccines against field challenge with Newcastle Disease Virus that could complicate bacterial respiratory diseases (i.e., APEC-related) appeared to be a common practice with 15 to 24% of flocks vaccinated at least once during the growing period.

Miscellaneous bacterial diseases of turkeys (data not shown) comprised of clostridial dermatitis, late-stage septicemia, staphylococcal tenosynovitis/osteomyelitis reportedly occurred, on average, 3% of flocks each year and no remarkable temporal changes in AMU for treating these diseases was noted. Of important note in the animal health data was the preventive use of nitarsone for histomoniasis in 2016 (4% of flocks).

##### Overall AMU, AMR and Flock Health Situation in Turkeys

As with broiler chickens and GF pigs, data from the turkey AMU questionnaire and the AMR data enabled assessment of the current AMU-AMR situation in the turkey sector. Total AMU trended downwards and corresponded with a significant decrease in multiclass resistant *E. coli* but relatively stable susceptible *E. coli*. Gentamicin resistance trended downwards but there were fluctuations in the levels of AMGL used during the study timeframe. High levels of CIP-R *Campylobacter* were detected despite limited FQ use. The diagnosis of enteric and respiratory diseases trended upwards but similar to broiler chickens, there were indications of transition from AMU-dependent preventive health approaches to non-AMU alternatives (vaccines, coccidiosis-focused enteric programs).

### 2.2. AMU and AMR Integration, Multispecies Summarized Analysis and Reporting

#### 2.2.1. AMU and AMR Summarized Reporting

**Figure 7:**
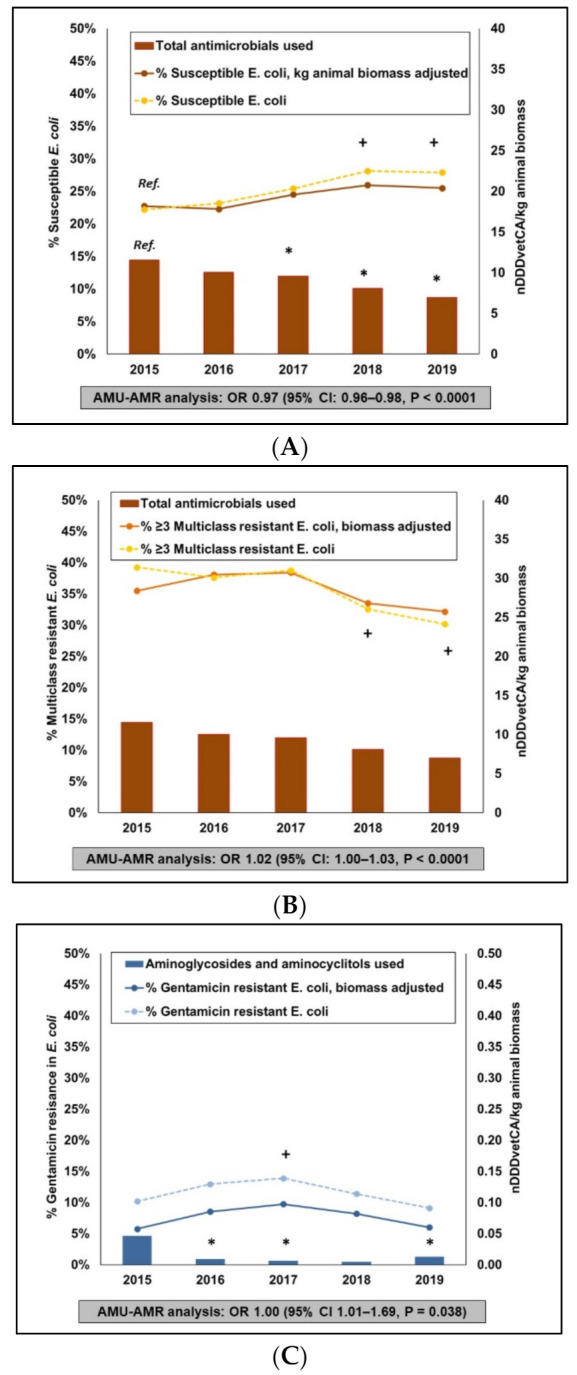

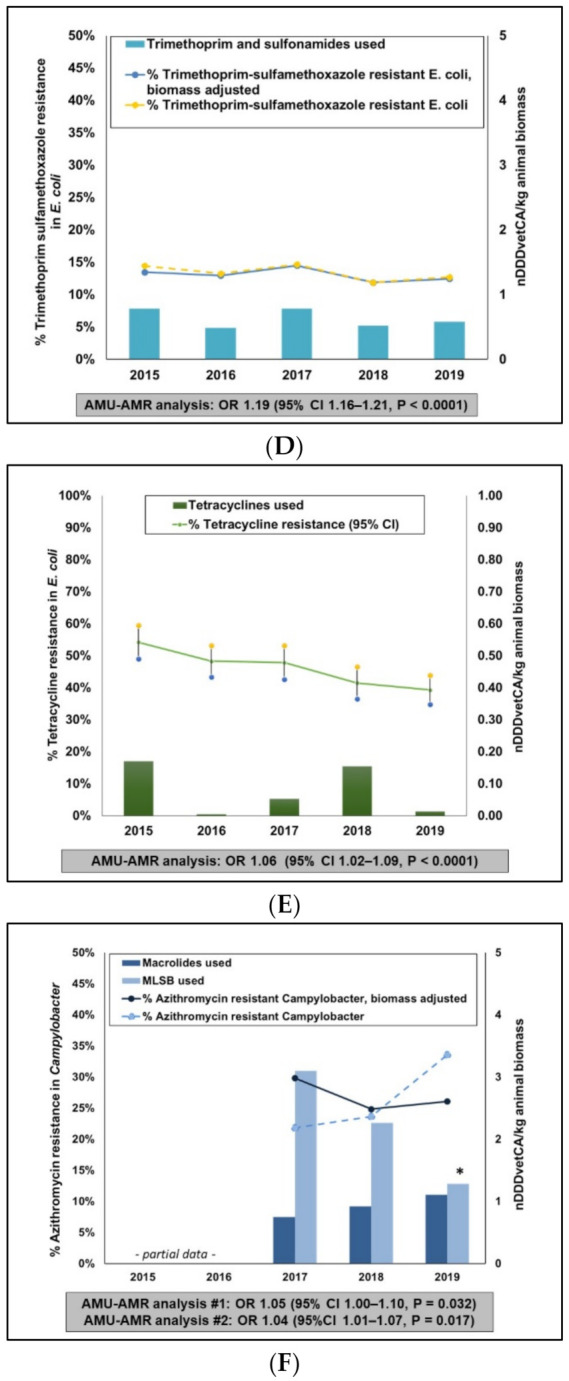
Summarized, multispeciesintegration of antimicrobial use and antimicrobial resistance, between 2015 and 2019. Two AMR indicators, percentage of resistance and percentage of resistance adjusted for animal biomass are shown. Total antimicrobials used—Susceptible (i.e., isolates that exhibited reduced or intermediate susceptibility to all antimicrobials tested) (**A**). Total antimicrobials used—Multiclass resistance (i.e., isolates that exhibited resistance to antimicrobials from at least three different antimicrobial classes) (**B**). Aminoglycosides and aminocyclitols used—Gentamicin resistance (**C**). Trimethoprim and sulfonamides used—Trimethoprim andsulfamethoxazole resistance (**D**). Tetracycline used—Tetracycline resistance (**E**). Analysis #1: Macrolides used—Azithromycin resistance and Analysis #2: Macrolides, lincosamides and streptogramin B use (MLSB)—Azithromycin resistance (**F**).* Significant (*p* ≤ 0.05) difference in AMU compared to the referent year (2015, labelled as Ref. in (**A**)). + Significant (*p* ≤ 0.05) difference in AMR compared to the referent year (2015). OR—Odds Ratio, 95%CI—95% confidence intervals. Data points for AMU are overall mean nDDDvetCA/kg animal biomass. Figure 7F contained partial data for 2015 (broiler chickens only) and 2016 (broiler chickens and turkeys). AMU-AMR associations were evaluated using two-level mixed effects logistic regression models.

This section synthesized the data from across the three species. This exercise was conducted to further refine the CIPARS approach for integration of farm-level AMU and AMR data across species for both present analyses and future expansion to other food producing animals. Its overall intent is to enable the reporting of summarized data to provide information on the overall AMU-AMR landscape in the Canadian food animal species. For this exercise, the eight AMU-AMR pairs were plotted (Figure 7). Upon visual inspection of the AMU-AMR data pairs, the trends in AMU paralleled the trends in AMR, except in the AZM-R and MACR use (or the alternate macrolide predictor variable MLSB).

##### Summarized, Multispecies AMU

As shown in Figure 7A, total nDDDvetCA/kg animal biomass decreased significantly (*p* < 0.0001) by 40% between 2015 and 2019. The use of World Health Organization’s (WHO) Highest Priority–Critically Important Antimicrobials (HP-CIAs) such as 3GCs and FQ was relatively low with <0.01 nDDDvetCA/kg animal biomass reported for either class (data not shown). Total AMGL and AMCL use (Figure 7C) significantly (*p* < 0.0001) decreased from 0.09 to 0.02 nDDDvetCA/kg animal biomass from 2015 to 2019. Other classes that trended downwards between 2015 and 2019 (Figure 7E,F) included TET (*p* = 0.002 from 1.9 to 0.59 nDDDvetCA/kg animal biomass) and MACR (*p* = 0.003, 1.70 to 0.11 nDDDvetCA/kg animal biomass) as well as MLSB (*p* < 0.0001, 3.05 to 1.29 nDDDvetCA/kg animal biomass).

##### Summarized, Multispecies AMR

The summarized AMR data using two AMR indicators, % resistance (adjusted for clustering at the species level) and the percentage of resistance adjusted for kg animal biomass are presented in Figure 7A–F. In most cases, both AMR indicators exhibited similar trends. However, the percentage resistance values were relatively higher in some cases (susceptible, GEN-R and AZM-R) while the kg animal biomass adjusted values were higher for TET-R.

A significant increase in % susceptible *E. coli* between 2015 and 2019 (*p* = 0.001, 22% to 28%) that corresponded with a significant decrease in multiclass resistant *E. coli* (*p* < 0.0001, 39% to 30%) was observed. This finding paralleled the trends observed in the individual species discussed in the previous sections. Other significant decreases in % resistance between 2015 and 2019 included CRO-R (*p* = 0.004, 7% to 4%), and TET-R (*p* = 0.021, 60% to 55%). No significant temporal trends were detected in the other homologous resistances studied (GEN-R, CIP-R and AZM-R).

##### AMU-AMR Associations

As summarized in the text boxes in Figure 7A–F, significant associations between AMU and AMR were detected in the composite AMR-AMU pairs, susceptible-total nDDDvetCA/kg animal biomass pair (OR 0.97, 95%CI, 0.96–0.98, *p* < 0.0001) and multiclass resistance–total nDDDvetCA/kg animal biomass pair (OR 1.02, 95%CI, 1.01–1.03, *p* < 0.0001). Associations in the homologous AMU-AMR pairs, specific to the antimicrobial agent/class were also significant for GEN-R-AMGL/AMCL use (OR 1.31, 95%CI, 1.01–1.69, *p* = 0.04), SXT-R-TMPS use (OR 1.19, 95%CI, 1.16–1.21, *p* < 0.0001) and TET-R-TET use (*p* = 1.08, 95%CI, 1.06–1.11, *p* = 0.000) for *E. coli*. Similarly, AZM-R in *Campylobacter* and MACR use (OR 1.04, 95% CI, 1.03 to 1.06, *p* < 0.000) were significantly associated. Associations between CRO-R in *E. coli* and 3GC use and CIP-R in *Campylobacter* and FQ use were not determined due to data limitations.

#### 2.2.2. Summarized Data Using Indicators Utilized in National and International AMU Surveillance Systems

To enable comparisons with international surveillance systems that are using the similar AMU indicators, overall AMU using the mg/PCU and mg/kg animal biomass were estimated using the multispecies combined data and shown in Table 1. The two weight-based indicators exhibited similar trends, but lower estimates for the mg/kg animal biomass. For the classes categorized by the WHO as HP-CIAs [31] or by Health Canada’s Veterinary Drugs Directorates (HC-VDD) as very high important (Category I) antimicrobial classes including 3GC’s and FQ’s [32], the overall uses were relatively low. The total AMU of these two antimicrobial classes combined contributed to <0.02% of the total AMU in 2019.

## 3. Discussion

This study built on CIPARS’ previous development of metrics and indicators, their utility for various AMU study objectives [26,27,28,29,30], and experience in integrating AMU and AMR surveillance data for routine reporting and communications [33]. For the first time, farm data were integrated from three animal species and animal health data were utilized to evaluate the current impact of AMU reductions in these food animal species. Regulatory changes to the Food and Drug Regulations in 2018 (Veterinary Drugs–Antimicrobial Resistance) resulted in enhanced veterinary oversight of medically important antimicrobials in an effort to contain AMR [18]. The voluntary industry AMU reduction strategies [19,20] aligned with these regulatory changes as well as the global recommendations to reduce AMU in animal production. It is envisaged that the structured approach for integration of AMU and AMR and animal health data will be progressively improved and eventually integrated into routine reporting and communication of CIPARS data. Ultimately, the goal of these data will inform broader livestock sector stewardship measures.

At the species level, the approach involved temporal analysis of the different data types followed by assessment of the strength of association between AMU and AMR of specified outcomes of interest. In this case, the approach for AMU-AMR-enabled synthesis of all available surveillance data and provided an overview of the AMU and AMR situation for that sector. The synopsis of surveillance data thus could be directed towards the relevant sector, from which AMU stewardship programs could be reviewed and further enhanced. As evidenced by the increasing trends in susceptible *E. coli* isolates, decreasing multiclass resistance and decreasing nDDDvetCA/kg animal biomass, the impact of AMU reduction and stewardship efforts, appear to be progressing across the three food animal species studied.

Integration of AMU and AMR surveillance data and the incorporation of animal health data are essential for informed sector-specific AMU stewardship and IPCs. Biosecurity variables have also been used in understanding AMU [3,34,35,36] and could be useful to add in the future. Under Canadian circumstances, a harmonized approach for collecting biosecurity status on farm and a scoring system, similar to work conducted elsewhere [37,38] could be explored for potential inclusion in the questionnaires.

The AMU-AMR pairs were assessed uniformly in all species but have greater relevance to specific species. For poultry, CRO-R and 3GC use, and CIP-R and FQ use, are important for monitoring the impact of Step 1 of the Canadian poultry AMU reduction strategy eliminating the preventive use of these classes. Following the implementation of Step 1 of the AMU strategy in May 2014 in the poultry industry, the data indicate that CRO-R *E. coli* and CIP-R *Campylobacter* spp. have persisted in poultry despite no reported use of 3GC’s and FQ, respectively. These findings are consistent with our earlier AMU/AMR surveillance findings from British Columbia [27] and other research [39,40] indicating that certain antimicrobial–bacterium combinations can persist in nature despite no or low antimicrobial selection pressure. The result of persistent AMR despite low or no AMU means that the impact of AMU reduction efforts may be variable. Another AMU-AMR pair of interest to the poultry industry was GEN-R and AMGL/AMCL use. The latter class of antimicrobials has been shown to co-select for GEN-R [41] and was therefore included in the GEN-R and AMGL/AMCL analysis. The relevant antimicrobials, GEN and lincomycin-spectinomycin were historically used at the hatchery level to prevent neonatal diseases of poultry. Compared to our earlier AMR surveillance data from British Columbia, the AMR outcome, GEN-R has decreased with decreasing use in the poultry industry but stable low levels of resistance continue to be detected. The elimination of the preventive use of AMGL/AMCL in the poultry industry constituted the 2nd step of the industry AMU reduction strategy, and was implemented at the end of 2018. Similar to 3GCs, a continued reduction in GEN-R prevalence may be observed in future years of surveillance. Lastly, the AZM-R and MACR use pair is important because AZM-R is highly correlated with erythromycin resistance. These outcomes are used as indicators for resistance to MACR, and broadly illustrate selection pressure linked to MACR and MLSB [42] which are antimicrobial classes commonly used in poultry and swine. Studies have repeatedly indicated an association between MACR use and MACR resistance in food animal species such as broiler chickens [43,44], pigs [45,46] and turkeys [44,47]. In this current study, the association was explored in two ways, first, between AZM-R and the MACR use alone, and secondly, between AZM-R and MLSB use. A significant association was observed in GF pigs for AZM-R and MACR and for AZM-R and MLSB pairs and a significant association was also noted between AZM-R and MACR in the multispecies analysis. These findings warrant further exploration of the underlying data, such as the proportion of *C. jejuni* and *C. coli* in the animal species surveyed, the relative contribution of the classes implicated for resistance to MACR or the broader group, MLSB. It is important to note that STRE (i.e., virginiamycin), which has been repeatedly identified as the most frequently used antimicrobial class in poultry [26,27,28,30] was no longer allowed for preventive use in both broiler [19] and turkeys [20] as part of Step 2 of the sectors’ AMU reduction strategy described above. As such, resistance to AZM-R and use of MLSB are both expected to decrease further beyond the surveillance timeframe used in this study. In addition to AMU, farm-level risk factors potentially contributing to the self-perpetuating cycle of antimicrobial-resistant *Campylobacter* (e.g., downtime and rest period, cleaning and disinfection) [48], which was observed in broiler chickens [49], also needs further investigation to determine if these factors are relevant in turkeys and GF pigs.

In poultry, the broad classification of diseases explored in this manuscript have been identified as risk factors for poor performance in Belgium, for example, neonatal septicemia led to increased mortality, and enteric diseases (coccidiosis infections, necrotic enteritis and dysbacteriosis) were associated with poor performance [50]. Our previous work highlighted that >80% of total AMU quantity (including ionophores and chemical coccidiostats) were intended for enteric diseases [30], thus the cost for the prevention of these diseases contributes substantially to the overall production input. Respiratory diseases (airsacculitis lesions) are also a major cause for condemnation in Canadian poultry [51]. Managing these diseases are crucial for the sustainability of the poultry industry. From the questionnaires, these disease syndromes were the frequently identified reasons for AMU. The animal health data and AMU trends have shown that the changing AMU patterns may explain, in part, the number of flocks with certain reported clinical syndromes. In poultry, a noteworthy downward trend in AMU at the hatchery level corresponded with an increase in the number of flocks reporting neonatal diseases. As for enteric diseases (typically a coccidiosis–NE concurrent infection) which can be multifactorial or multi-etiologic in nature, there was evidence of a slight increase in flocks that reportedly experienced enteric diseases but AMU intended for enteric diseases (e.g., BAC, PEN, STRE) decreased compared to our earlier data (26–27). However, it appears that there are options available to reduce AMU while still managing enteric disease. For example, for farms transitioning to RWA production, these farms are allowed to use chemical coccidiostats which may help control enteric disease. Reduced AMU programs also may involve the use of ionophores alone, thereby indicating that controlling coccidiosis could potentially offset the need for AMU towards NE, in particular, those that are medically important antimicrobials. The ongoing monitoring of these diseases are important in routine surveillance for providing context, in particular, the circumstances in which AMU was required. As documented in other surveillance reports, even a single health event could shift the national consumption of antimicrobials for a specific sector [52].

Diseases of economic importance to the Canadian swine industry include *E. coli, Hemophilus parasuis, Lawsonia* spp., *Streptococcus suis* and Swine Influenza Virus and its sequelae (e.g., secondary bacterial infections), due to both decreased growth rates and increased mortality. The availability of efficacious vaccines can impact the use of antimicrobials to treat and control these diseases. For example, in this study, the significant increase in the use of *Lawsonia* vaccines corresponded with a significant decrease in antimicrobial use for *Lawsonia*, however a significant increase in farms reporting *Lawsonia* was also observed. In order to further understand these relationships, additional data on the level and duration of antimicrobial use for specific disease situations, morbidity rates, and timing of vaccinations would be useful. Although the collection of this detailed data may be limited to a research setting in order to preserve the practicality and sustainability of the surveillance system, the integration of this health and AMU data provides direction for such investigations.

The lack of efficacious vaccines can substantially impact antimicrobial use in swine. For example, *Streptococcus suis* significantly impacts animal health and production in Canada, but a commercial vaccine is not available and *Streptococcus suis* is consistently one of the most common diseases identified in the CIPARS questionnaires, where antimicrobials are used for treatment and control. For other diseases, such Swine Influenza Virus and *E. coli* the number of different strains of the disease agent and a lack of cross-protection in existing vaccines, can limit the usefulness of vaccines in many farms. New vaccine technologies and regional approaches to vaccine development may address some of these limitations. The integration of AMU and animal health information provides an opportunity to monitor changes in vaccine use, when new vaccines become available.

The final step of the analysis combined data from all the species. This approach is useful for monitoring the overall AMR in relation to total AMU in the food animal sector. The AMU component involved summation of AMU across the three species. For this exercise, the AMU indicator should be similar to those that are used by other national and international surveillance systems to enable comparisons. The summarized data indicated that total AMU in mg/PCU decreased compared to 2015 levels; the baseline for this study. Overall, there has been a simultaneous drop in the majority of the classes commonly used in the three animal species studied. This was consistent with the national sales and distribution data which decreased from 183 to 149 mg/PCU between 2015 and 2018 [53]. When compared with the global AMU data collected by the OIE, the AMU across these three food animal species measured in mg/kg animal biomass (combined data at 57 mg/kg animal biomass) was relatively lower compared to those reported for the Americas for that year (90.50 mg/kg animal biomass, adjusted by reported regional coverage) [24]. It is important to note that these two weight-based indicators exhibited the same trend which was consistent with our previous findings using data from poultry [28].

The AMR outcomes using the combined data from the three species exhibited a modest increase (susceptible) or decrease (multiclass resistance) that mirrored the trends seen in the species-specific data. These two composite indicators are complementary, used as primary and secondary AMR outcomes, respectively, or Key Outcome Indicators for AMR [54,55]. One of these AMR outcomes could be used for routine integration, however, at this point when the animal species are in transition towards reduced AMU production, these two AMR outcomes could provide an indication on how the population of bacteria are shifting (e.g., from a population of bacteria with multiclass resistance towards a more susceptible bacterial population). Shift from multiclass resistance towards homologous resistance could also be monitored during these transition period towards reduced AMU in food animal production. Of important public health concern is the continued increase in CIP-R. Trends in other AMU-AMR pairs studied appeared to be decreasing (GEN-R and AMGL/AMCL use, SXT-R and TMPS use, TET-R and TET use) or stable (CRO-R and 3GC use).

## 4. Methods

Our previous works on AMU metric and indicator development, familiarity with the attributes of the different AMU indicators [28] and AMR outcomes [27], and the evaluation of the utility of indicators appropriate for certain AMU study objectives (this current study) informed the development of a structured approach for AMU/AMR data integration, a refinement of current methods (largely descriptive) for surveillance data integration [53,56]. A summary of relevant works including various study timeframes and data components used in this current study, is referenced throughout this paper. In brief, the methodology for AMU-AMR data integration involved descriptive and temporal analysis of AMU/AMR data according to routine CIPARS methods [53,56], synthesis of results, selection of AMU-AMR pairs (e.g., outcomes based on public health, animal health or general outcomes with relevance to stewardship monitoring), followed by AMU-AMR analysis using modelling approaches, and data visualization of the combined data. An exploratory AMU indicator, nDDDvetCA/kg animal biomass, a derivative of nDDDvetCA/PCU previously used [28] was utilized in AMU-AMR association analysis.

In brief, the following outlines the approach:

### 4.1. AMU

Broiler chicken (2015 to 2019), grower–finisher swine (2015 to 2019) and turkey data (2016 to 2019) were obtained from commercial broiler chicken and turkey flocks (quota holding producers with >1000 birds at any given 8 week grow-out period; conventional, RWA and organic flocks included but excluding free-range flocks) and commercial grower finisher swine herds (>1000 heads sent for slaughter in the sampling unit per year, excluding organic and pasture-raised herds) using species-specific questionnaires administered by the veterinarian or a designate to their producers. Data were entered into the CIPARS PostGRESQL database (i.e., farm-level AMU surveillance data repository) and validated as described elsewhere [28,56]. Animal health and AMU dichotomous outcomes, quantitative AMU and farm demographics data were extracted into Microsoft Excel (Microsoft Office Professional Plus 2016), checked for errors and analyzed. Flock and herd level descriptive statistics were obtained including the mean, median, and 95% confidence intervals (CI) for total AMU or class-specific (homologous) AMU.

### 4.2. AMR

Data were extracted from the Public Health Agency of Canada (PHAC) AMR data repository (DEXA) followed by data validation and analysis. In brief, using Sensititre microbroth dilution technology, and the National Antimicrobial Resistance Monitoring System (NARMS) Gram-negative CMV4AGNF and CAMPY panel, prevalence of homologous resistances (i.e., resistance of an isolate to a single antimicrobial agent) were determined for *E. coli* and *Campylobacter*, respectively [56]. In addition, two additional AMR outcomes, multiclass resistance (i.e., isolates that exhibited resistance to antimicrobials from at least three different antimicrobial classes included in the above named NARMS panels) and susceptible isolates (i.e., isolates classified as susceptible or intermediate to antimicrobials included in the above named NARMS panels) were determined. In total, eight AMR outcomes were used in data integration.

### 4.3. Methodology for Species-Level Integration and Analysis

Initially, AMU and AMR results were visually inspected, examined for significant temporal changes and relevant findings were synthesized. Subsequently, the AMU indicator appropriate for comparing temporal trends and the levels of AMU between species was selected from five candidate AMU indicators comprised of two weight-based (mg/population correction unit [mg/PCU] and mg/kg animal biomass [mg/kg animal biomass]) and three dose-based indicators (nDDDvetCA/1000 animal days at risk, nDDDvetCA/PCU, nDDDvetCA/kg animal biomass). The selection of the most appropriate indicator to meet the current study objectives was based on a systematic evaluation of the above listed AMU indicators. One of the key factors in deciding on the best metric to use included the ability to be able to compare AMU between species. To do this it was important to have an adjustment for defined daily doses per antimicrobial by species, as well as an adjustment for animal population and weights. It was also important to identify an indicator that would be familiar to our producer and veterinarian stakeholders. As a result, kg animal biomass was determined to be the better fit than the PCU denominator as this can translate into the amount of antimicrobial to raise and animal to slaughter weight. The kg animal biomass denominator was also favorable to the PCU denominator because there is a reduction in the analytic burden when weight at treatment (used in the PCU estimation) is unavailable or difficult to obtain. The final benefit of the kg animal biomass is that in the future it can be easily replicated in other species such as aquatic animals. For all of the above reasons the most appropriate indicator to meet the current study objectives was the nDDDvetCA/kg animal biomass. This exploratory indicator which is used in this current study and in a previous analysis [28] is similar to nDDDvetCA/PCU previously used by CIPARS [28] and pertains to the total or class-specific nDDDvetCA (mg adjusted by the animal-specific DDDvetCA standard) used in every kg of live animal pre-slaughter weight. This measurement is comparable to the annualized reporting of species-specific trends and levels of AMU in the Netherlands (DDDA/year) [4,57]. The AMU and AMR outcomes were then determined and included six AMR outcomes for *E. coli* and three for *Campylobacter*, paired with either the total or class-specific AMU outcomes. The AMR in *E coli* and relevant AMU included: (1) Susceptible isolates and total AMU, (2) Multiclass resistance and total AMU, (3) CRO-R and 3GC’s use (4) GEN-R and AMGL use including AMCL use, (5) SXT-R and TMPS use, and (6) TET-R and TET use. The AMR in *Campylobacter* and corresponding AMU included: (1) CIP-R and FQ use (2) AZM-R and MACR use, and (3) AZM-R and the MLSB use. The last pair which could be used as an alternate to number 2, was of interest because of potential co-selection to MACR in *Campylobacter* among these commonly used classes of antimicrobials in the species examined [58,59]. These AMU-AMR outcome combinations are those used for harmonized monitoring in other surveillance systems (i.e., susceptible isolates or multiclass resistant isolates and total AMU) [12,54], have an elevated public health importance (e.g., CRO-R and 3GC’s use, CIP-R and FQ use), or have moderate to high public health and veterinary importance.

The CIPARS questionnaires collect data on general farm or site information, biosecurity, animal inventory, AMU and health status. The animal health status component of the questionnaires collects information about the disease status of the flock or herd, based on the clinical impression of the veterinarian (e.g., as entered in flock or herd health records as per on-farm food safety program, confirmation with the producer/farm staff, post-mortem findings, laboratory confirmation), vaccines administered and other preventive health programs relevant for the disease or etiologic agent (e.g., nonantimicrobial alternatives or approaches). Temporal trends in the frequency of flocks or herds deemed positive or likely positive to the disease syndrome/ etiologic agent were also assessed to determine if the shifts in AMU were correlated with the occurrence of disease or prompted the use of non-antimicrobial interventions.

### 4.4. Methodology for Multispecies Integration and Analysis

Data from the three species (broiler chickens, GF pigs and turkeys) were combined for the purposes of summarized reporting. For AMR, the overall prevalence of resistance was determined by taking the total number of isolates that were resistant for all three species and dividing by the total number of isolates recovered from the three species. Prevalence of resistance was then adjusted by the overall biomass for all three species to provide an outcome similar to AMR Indicator Index or Key Outcome Indicator consistent with the literature [12,54,55]. For this study, instead of adjusting AMR for PCU, the AMR data were adjusted for the actual kg pre-slaughter weights (i.e., collected at the time of farm visit corresponding to the period closest to the estimated slaughter date). As for the AMU component, the nDDDvetCA/kg animal biomass was used for overall AMU-AMR association analysis. For comparison of the overall levels of AMU with other surveillance systems, the general AMU indicator used by CIPARS, milligrams per population unit (mg/PCU) developed by the European Surveillance for Veterinary Antimicrobial Consumption (ESVAC) and the exploratory indicator mg/kg animal biomass, the indicator used by the OIE in reporting the global AMU data were used to summarize the AMU data from the 3 species. The summarized values (total mg/total PCU; mg/total kg animal biomass) were determined. The purpose of this exercise was to compare the levels of the total AMU in the combined/multispecies AMU data to the most recent ESVAC, OIE reports [23,24] and the CIPARS national sales and distribution data [53].

### 4.5. Analysis

All analyses were performed in Stata SE V16.1 (College Station, Texas) and Microsoft Excel (Microsoft Office Professional Plus 2016). For temporal analysis, trends were descriptively assessed (e.g., percent change) followed by logistic regression analysis. Models were built with relevant data (e.g., AMU count-based indicator, AMR and diagnosis of diseases and certain animal health information) as binary outcome variables and year as independent categorical variable (i.e., LOGISTIC procedure in Stata SE/V16) and *p* ≤ 0.05 was considered significant. In general, 2015 was the referent year used in all models. In certain situations, notable changes between two time points other than the study timeframe (2015 and 2019) were described.

As for the quantitative AMU data, the flock or herd distributions were visually inspected and descriptive statistics were obtained (% change between referent year and subsequent years, mean, median, 95% CI). Due to the skewed distribution of the flock or herd-level AMU data (similar in all species), temporal changes were determined using nonparametric Wilcoxon rank sum test between two time points.

For studying the relationships between the nine pairs of AMU (nDDDvetCA/kg animal biomass as the continuous predictor variable) and AMR (isolate resistant or susceptible as the binary outcome variable) outcomes, mixed effects logistic regression models (MELOGIT procedure in Stata SE/V16) were fitted for each species, adjusting for year of study with random effects for flock or herd in order to account for similarities in AMR of multiple isolates within a flock or herd. Only antimicrobial classes with values ≥ 0.1 nDDDvetCA/kg animal biomass were modelled to achieve model convergence and to obtain reliable effect estimates (OR, 95% CIs and level of significance). The same modelling approach was used for the multispecies data, adjusting for year of study with random effects for species and flocks or herds (i.e., two-level random slope models).

For data visualization, AMU and AMR data were plotted in Microsoft Excel. Similarly, the count-based AMU and animal health outcomes (diseases diagnosed, animal health information) were plotted and visually inspected for similarities in trends which provided some context on the potential animal health implications of the changes in AMU. The percentages of mortality by species were also determined as additional information.

## 5. Conclusions

The methodology for AMU-AMR data integration presented in this paper evolved from previous analytic exercises, exploratory works on the use of different AMU indicators using input parameters derived from AMU surveillance data, and current knowledge and experiences by other surveillance systems on AMU-AMR integrated analysis and reporting. The high-resolution data collected by CIPARS at the farm level enabled integration and evaluation of exploratory analysis for routine surveillance reporting while the syndromic data provided an indication of the potential animal health implications of the changing AMU practices in Canadian poultry and swine sectors. Notable decreases in AMU and AMR in commonly used antimicrobial classes and total AMU (nDDDvetCA/kg animal biomass) provide early indications of the positive impact of stewardship policy in these food animal sectors in Canada. Future surveillance data synthesis and integration will continue to provide valuable feedback on the progress of AMU interventions. For example, the magnitude of the decrease in AMU/AMR levels 3 to 5 years following the full implementation of the AMU reduction initiative in the poultry industry (Step 3). Ongoing analysis of the species-specific and combined data and summarized reporting will also facilitate in-depth analysis of potential risk factors for AMR across all food animal species and potentially inform sector-level or food animal industry-wide interventions. However, to fully complement the current integrated AMU and AMR findings and to support ongoing stewardship the inclusion of clinical pathogens and Gram-positive AMR indicators will be necessary.

## Figures and Tables

**Table 1:** Summarized analysis of antimicrobial use using weight-based indicators from broiler chickens, grower–finisher pigs and turkeys between 2015 and 2019.

	Year	2015	2016	2017	2018	2019
**AMU Indicator**	**Number of Farms**	**250**	**299**	**293**	**333**	**352**
**mg/PCU**	Total antimicrobials	175	106	104	106	109
	3rd generation cephalosporins	0	0	0.009	0.016	0.010
	Fluoroquinolones	0	0	0.005	0.004	0.013
**mg/kg animal biomass**	Total antimicrobials	91	59	57	58	59
	3rd generation cephalosporins	0	0	0.005	0.009	0.005
	Fluoroquinolones	0	0	0.003	0.002	0.007

## Data Availability

Data are summarized in the tables, figures, Supplementary S1–S5 and Part I of this study (Methodology Development).

## Supplementary Materials

Additional information are available online at https://www.mdpi.com/article/10.3390/pathogens10111491/s1.
