# Supplementary material for: Informing Stewardship Measures in Canadian Food Animal Species through Integrated Reporting of Antimicrobial Use and Antimicrobial Resistance Surveillance Data—Part II, Application
Source: Pathogens. 2021 Nov 16;10(11):1491. doi: 10.3390/pathogens10111491 (PMC8621420; doi:10.3390/pathogens10111491)
Supplement: Supplementary file 1 [file pathogens-10-01491-s001.zip › pathogens-1385822-SI.pdf]

Supplementary Materials

Supplementary Materials S1. Most frequently occurring antimicrobial resistance (class) patterns (Supplement to AMR findings)

Table S1. Broiler chickens, 2015 to 2019.

AMR (class) Profiles	Number of isolates
**AMIN-FOL-TET-**	**278**
**AMIN-BLA-FOL-TET-**	**237**
**TET-**	**173**
**BLA-**	**153**
**AMIN-FOL-**	**112**
**AMIN-BLA-**	**106**
**AMIN-BLA-TET-**	**102**
**BLA-TET-**	**102**
**AMIN-TET-**	**98**
**AMIN-BLA-FOL-**	**76**
AMIN-BLA-FOL-PHE-TET-	70
AMIN-	61
FOL-	46
AMIN-FOL-PHE-TET-	34
QNL-	34
FOL-TET-	31
BLA-FOL-TET-	28
AMIN-BLA-FOL-QNL-TET-	25
AMIN-FOL-QNL-TET-	21
BLA-FOL-	21
AMIN-BLA-QNL-	18
AMIN-BLA-FOL-PHE-	16
AMIN-QNL-	16
AMIN-FOL-PHE-	15
BLA-QNL-	10
FOL-PHE-TET-	10
FOL-QNL-	9
AMIN-BLA-FOL-MACR-TET-	8
AMIN-BLA-FOL-QNL-	8
AMIN-QNL-TET-	8
AMIN-BLA-QNL-TET-	7
BLA-QNL-TET-	7
QNL-TET-	6
AMIN-FOL-QNL-	5

AMIN-FOL-QNL-PHE-TET-	4
AMIN-BLA-FOL-QNL-PHE-TET-	3
BLA-FOL-PHE-TET-	3
BLA-FOL-QNL-TET-	3
BLA-PHE-TET-	2
AMIN-PHE-TET-	1
BLA-FOL-PHE-	1
MACR-TET-	1
National	1969

Top 10 most frequently occurring profiles are in bold fonts.

Table S2. Grower-finisher pigs, 2015 to 2019

AMR (class) Profiles	Number of isolates
**TET-**	**467**
**AMIN-FOL-TET-**	**246**
**AMIN-TET-**	**221**
**AMIN-BLA-TET-**	**161**
**BLA-TET-**	**137**
**AMIN-BLA-FOL-PHEN-TET-**	**134**
**AMIN-BLA-FOL-TET-**	**111**
**AMIN-FOL-PHEN-TET-**	**111**
**FOL-TET-**	**83**
**BLA-**	**76**
BLA-FOL-TET-	61
BLA-FOL-PHEN-TET-	60
AMIN-	59
AMIN-FOL-	39
FOL-PHEN-TET-	33
AMIN-FOL-PHEN-	20
AMIN-BLA-	13
FOL-	13
FOL-PHEN-	13
AMIN-BLA-FOL-	12
AMIN-BLA-FOL-PHEN-	12
BLA-FOL-	10
BLA-FOL-PHEN-	9
AMIN-BLA-FOL-PHEN-MACR-TET-	4
AMIN-FOL-MACR-TET-	3
AMIN-BLA-FOL-QNL-PHEN-TET-	2
AMIN-BLA-FOL-QNL-TET-	2
BLA-PHEN-TET-	2

BLA-QNL-TET-	2
PHEN-	2
PHEN-TET-	2
QNL-	2
QNL-TET-	2
AMIN-BLA-FOL-MACR-TET-	1
AMIN-BLA-QNL-TET-	1
AMIN-FOL-QNL-MACR-TET-	1
AMIN-PHEN-TET-	1
BLA-FOL-MACR-TET-	1
BLA-PHEN-	1
FOL-MACR-TET-	1
National	2131

Top 10 most frequently occurring profiles are in bold fonts.

Table S3. Turkeys, 2016 to 2019

AMR (class) Profiles	Number of isolates
**TET-**	**170**
**AMIN-TET-**	**150**
**AMIN-BLA-FOL-TET-**	**130**
**AMIN-FOL-TET-**	**96**
**AMIN-BLA-TET-**	**93**
**BLA-TET-**	**55**
**FOL-TET-**	**41**
**BLA-**	**29**
**AMIN-BLA-**	**28**
**BLA-FOL-TET-**	**23**
AMIN-FOL-	20
FOL-	19
AMIN-FOL-PHEN-TET-	18
AMIN-	15
AMIN-BLA-FOL-	14
AMIN-BLA-FOL-PHEN-TET-	13
BLA-FOL-	7
AMIN-FOL-QNL-TET-	4
QNL-	4
AMIN-BLA-FOL-QNL-TET-	3
BLA-PHEN-TET-	3
PHEN-TET-	3
AMIN-BLA-FOL-MACR-TET-	2
AMIN-BLA-PHEN-TET-	2

AMIN-BLA-QNL-TET-	2
BLA-QNL-PHEN-TET-	2
FOL-QNL-TET-	2
AMIN-BLA-FOL-PHEN-	1
AMIN-BLA-FOL-QNL-PHEN-TET-	1
AMIN-BLA-PHEN-	1
AMIN-BLA-QNL-	1
AMIN-FOL-QNL-	1
BLA-FOL-PHEN-	1
BLA-FOL-PHEN-TET-	1
BLA-FOL-QNL-PHEN-TET-	1
BLA-QNL-TET-	1
FOL-PHEN-	1
FOL-PHEN-TET-	1
National	959

Top 10 most frequently occurring patterns are highlighted in bold fonts.

Supplementary Materials S2. Percentage of mortality in the animal sectors studied between 2015 and 2019, Supplementary to animal health

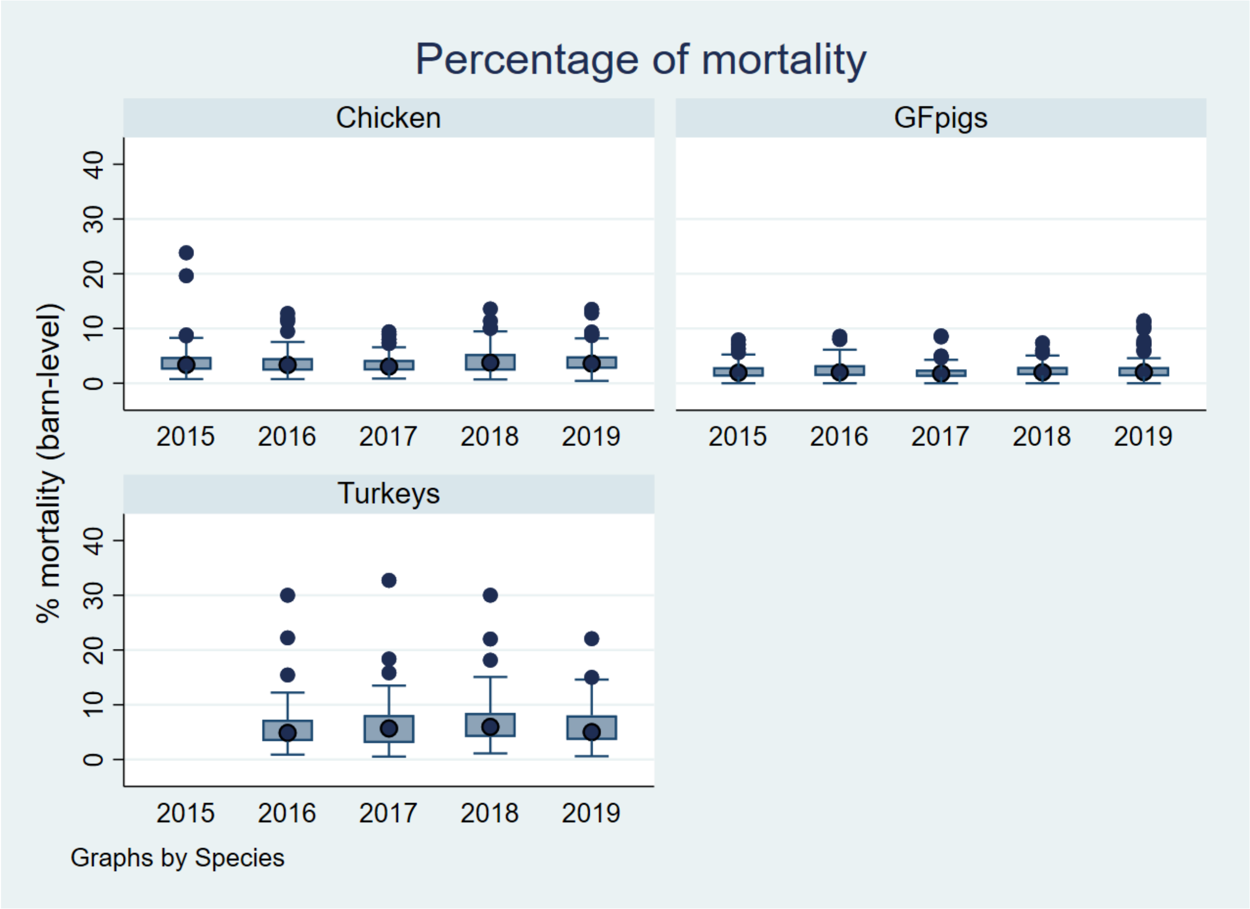

Supplementary Materials S3. Enteric programs in broiler chickens, supplementary to Figure 2.B (enteric diseases).

Figure S1. Coccidiosis programs

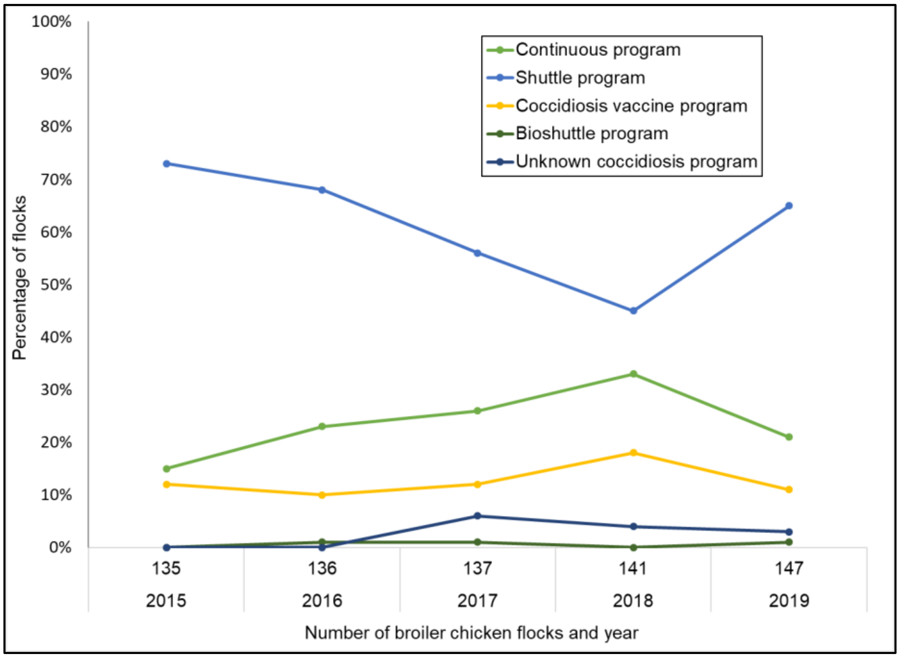

Figure S2. Necrotic enteritis programs

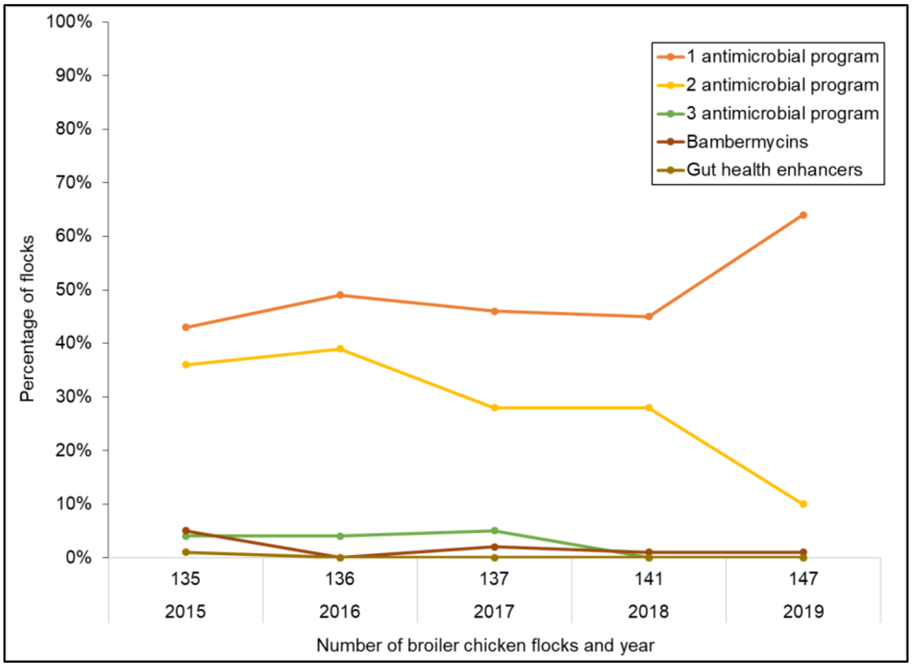

Please note that partial data presented above (2015-2017) were reported in another study[1].

[1] ***Agunos A,*** Deckert A, Leger D, Gow S, Carson C. Antimicrobials Used for the Therapy of Necrotic Enteritis and Coccidiosis in Broiler Chickens and Turkeys in Canada, Farm Surveillance Results (2013-2017). Avian Dis (2019) 63:433-45 doi: 10.1637/11971-091718-Reg.1.

Supplementary Materials S4. Enteric programs in turkeys, supplementary to Figure 6.B.

Figure S3. Coccidiosis programs

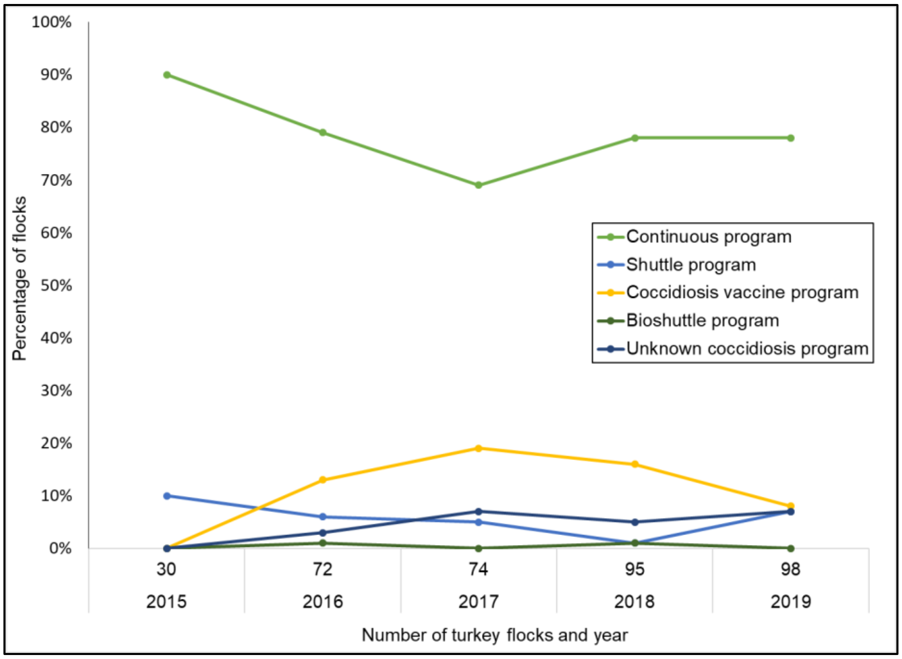

Figure S4. Necrotic enteritis programs

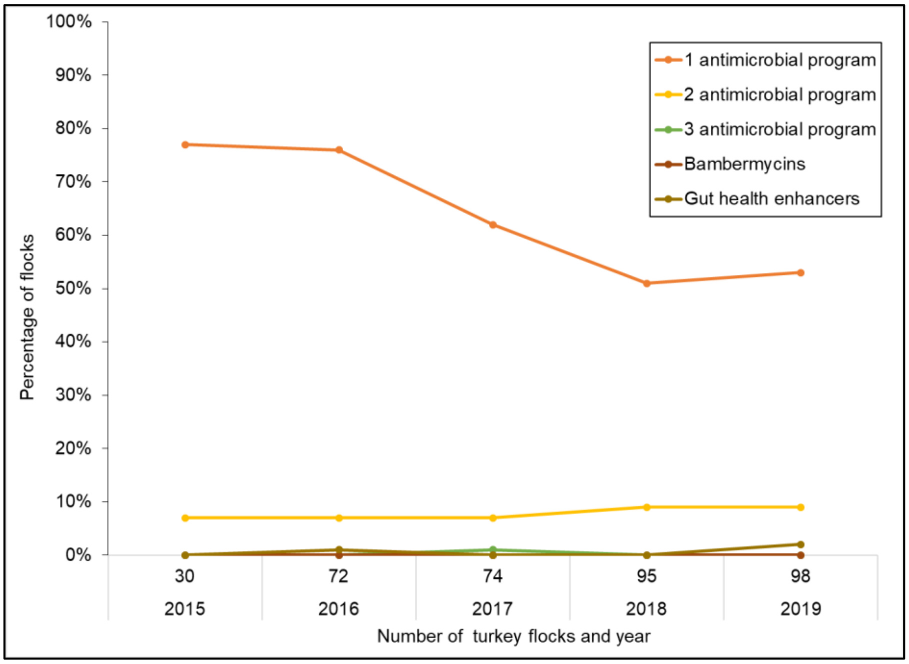

Please note that partial data presented above (2015-2017) were reported in another study[2].

[2] ***Agunos A,*** Deckert A, Leger D, Gow S, Carson C. Antimicrobials Used for the Therapy of Necrotic Enteritis and Coccidiosis in Broiler Chickens and Turkeys in Canada, Farm Surveillance Results (2013-2017). Avian Dis (2019) 63:433-45 doi: 10.1637/11971-091718-Reg.1

Supplementary Materials S5. Synthesis of studies that formed the basis for AMU-AMR integration methodology development.

	1st *in:* Plos One	2nd- *in:* Frontiers, 2019	3rd *in:* Frontiers, 2020	4th This current study*
Timeframe	2013 to 2015	2013-2017	2015-2019	2015-2019
Data coverage	National data – 4 (2013) to 5 provinces (2014 and 2015)	British Columbia FoodNet Canada sentinel site only	National data – 5 provinces	National data – poultry and swine producing regions/provinces
Species	Broilers only	Broiler<br>Turkeys	Broilers<br>Turkeys	Broilers<br>Grower finisher pigs<br>Turkeys<br>Combined multispecies
Data components	AMU only; includes coccidiostats	AMU<br>AMR	AMU only, medically-important antimicrobials	AMU<br>AMR<br>Animal health
AMU surveillance objectives and indicators	• **Describe early AMU data collected (3 years)**<br>• **Trends over time**<br>• **Compare with national sales and distribution and grower-finisher pigs annual quantity of use.**<br><br>***Indicators:***<br>• Count-based: frequency<br>• Weight-based: mg/PCU<br>• Dose-based: nDDDvetCA/1,000 chicken-days at risk	• **Trends over time.**<br>• **How trends in AMU relate to AMR findings**<br>• **Methods development (use of summarized indicator) to inform integrated analysis and reporting (multispecies) for national level analysis**<br><br>***Indicators:***<br>• Count-based: frequency<br>• Weight-based: mg/PCU<br>• Dose based: nDDDvetCA/1,000 animal-days at risk, nDDDvetCA/PCU	• **Describe various AMU parameters**<br>• **Comparison of AMU levels between flocks (an approach similar to "benchmarking")**<br>• **Comparison between poultry species**<br>• **Methods development: for flock or national level reporting (inform data visualization)**<br><br>***Indicators:***<br>Count-based: frequency (updated from the 1st paper).<br>Using the denominators used in the 2 previous papers:<br>• mg/PCU vs. *mg/kg animal biomass*<br>• nDDDvetCA/1,000 animal days at risk, vs. *nDDDvetCA/1,000 kg animal days at risk*<br>• nDDDvetCA/PCU vs. n*DDDvetCA/kg animal biomass*	• **Comparison of AMU levels and temporal trends between animal species.**<br>• **Comparison of AMU with national (sales and distribution data) and internationally (ESVAC and OIE).**<br>• **Investigating AMU and AMR association in pairs that have relevance to public and animal health in Canada and general stewardship outcomes.**<br>• **Monitor impact of AMU stewardship actions (STEPS 1 and 2 in the poultry industry and regulatory changes in veterinary AMU) in terms of AMU and animal health.**<br><br>***Indicators depending on the study objective:***<br>1) AMU-AMR association modelling exercises, species-level analysis and multispecies analysis: nDDDvetCA/kg animal biomass<br>2) AMU-animal health: count based indicators.<br>3) Comparisons with other surveillance programs:<br>• mg/PCU (national data, ESVAC)<br>• mg/kg animal biomass (OIE)
AMR surveillance indicator	N/A	% Resistance<br>AMR Indicator Index **(% resistance adjusted for PCU**** across poultry species in the sentinel site)	N/A	% Resistance<br>**AMR adjusted for kg animal biomass ****(% resistance adjusted for kg animal biomass in the 3 species surveyed, modified from AMR Indicator Index, study #2).

Methodology	Methods highlighted the feed and water estimation using growth curves for the breed (Ross and Cobb, local guidelines)	Explored summarized AMR indicator as per JIACRA II AMR indicator index and characterized the primary (susceptible E. coli) and secondary (multiclass resistant E.coli) and select homologous AMR outcomes (ceftriaxone/ceftiofur, gentamicin, ciprofloxacin)<br>AMU-AMR pairs were largely based on their relevance to public health.	Explored how the AMU indicators relate to each other (weight vs. dose-based) using pairwise comparisons<br>Explored flock distribution of AMU using multiple metrics and indicators and descriptive statistics<br>Identified high and low users of antimicrobials using modelling approaches.	Structured integration of various data components and evaluated the utility of animal health data for context.<br>AMU-AMR pairs analyzed were based on their relevance to overall stewardship actions, public health and animal health.
Analysis	**NATIONAL-level** summaries (aggregated sum if quantitative or prevalence measures if binary)<br>Descriptive and temporal<br>Seasonality of use<br>Reasons for use	**PROVINCE-level** summaries (aggregated sum if quantitative data or prevalence measures if binary)<br>Descriptive only: assessed trends of the AMU-AMR pairs and characterized relative changes.<br>**Not done: AMU-AMR association analysis**	**FLOCK-LEVEL** descriptive statistics (mean, median, range).<br>Between flock variations and how AMU indicators relate to each other<br>(pairwise comparisons)<br>Identification of high users of AMU (logistic regression models)<br>NATIONAL-LEVEL visualization and trends updated from the first paper,	**FLOCK and HERD LEVEL** descriptive analysis and temporal trends.<br>**More structured AMU-AMR data integration:** Investigation of AMU and AMR using mixed effects logistic regression analysis.<br>Temporal analysis of AMU methodology: using nonparametric tests in addition to descriptive assessment (relative change).<br>List of AMR outcomes expanded from 2nd paper to include resistances to the following: tetracycline, trimethoprim and sulfamethoxazole and macrolides which were used by high user flocks in the 3rd paper.<br>Animal health trends (diseases diagnosed, preventive health variables).
Highlights/unique findings	Early data indicated high mg/PCU (142 to 153)<br>Early indication of the success of the poultry industry's voluntary AMU strategy eliminating the preventive use of certain antimicrobials (STEP 1 – 3rd generation cephalosporins.	**Species-specific analysis:** Trends in AMU frequency paralleled the trends in AMR prevalence.<br>**Summarized reporting (AMR indicator indices):** reflected the individual species trend except for fluoroquinolone resistance and fluoroquinolone use pair where resistance continued to be detected with low reported use.<br>Unintended consequence of the AMU reduction policy detected (e.g., increase in gentamicin and lincomycin-spectinomycin use with corresponding increase in gentamicin resistance).	High users of antimicrobials in both chickens and turkeys were those that used trimethoprim-sulfonamides, bacitracins, and tetracyclines. Flocks that used certain classes were more likely to be in the top 25th percentiles of users of certain classes (e.g., aminoglycosides and penicillins in broilers, penicillins in turkeys)<br>The dose-based indicators were highly correlated;weight-based and dose-based indicators were moderately correlated.	Mg/PCU decreased to **109** compared to early data (Study #2).<br>Significant associations detected in certain homologous AMU-AMR pairs.<br>Negative association between susceptible isolates and total AMU, an early indication that AMU reduction strategies in the food animal sectors are having an impact.<br>Increase in the reported occurrence of disease syndromes/etiologic agents highlighted the need for ongoing monitoring of animal health parameters in addition to AMU and AMR.

Suggested further analysis		Application of the methods to national data and multispecies integration.	Determine if high users (or use in general) of certain classes are linked to AMR,	This approach informed future integrated analysis and reporting.<br>Expansion of the AMU indicator used here (nDDDvetCA/kg animal biomass) in other species.

*Part I of this study describes the approach for integration of antimicrobial use and antimicrobial resistance data.

**Similarly adjusted the resistance data by the population and weight of the animals studies: population correction unit or kg animal biomass based on actual live preslaughter live weights collected closest to the approximate slaughter date.
